# Hierarchical Star–Sphere ZnCo_2_O_4_/Graphene Oxide/Pt Nanocomposites for Low-Temperature Hydrogen Sensing

**DOI:** 10.3390/s26165255

**Published:** 2026-08-19

**Authors:** Hussein A. Younus, Zeyana Al Shueili, Zivar Azmoodeh, Mohammed Al Abri, Rashid Al Hajri, Hassan Al Lawati

**Affiliations:** 1 Nanotechnology Research Centre, Sultan Qaboos University, Muscat 123, Oman; s130660@student.squ.edu.om (Z.A.S.);; 2 Department of Petroleum and Chemical Engineering, College of Engineering, Sultan Qaboos University, Muscat 123, Oman; 3 Department of Electrical and Computer Engineering, College of Engineering, Sultan Qaboos University, Muscat 123, Oman

**Keywords:** hydrogen sensing, ZnCo_2_O_4_ spinel, star–sphere architecture, graphene oxide, Pt sensitization, morphology engineering, low-temperature gas sensing

## Abstract

Hydrogen (H_2_) detection under practical operating conditions requires sensing materials that simultaneously provide accessible reaction sites, efficient gas diffusion pathways, and fast interfacial charge transfer. Here, a hierarchical star-sphere ZnCo_2_O_4_ (ZC) architecture was integrated with graphene oxide (GO) and Pt supported on graphitized carbon (Pt/C) to develop hybrid chemiresistive sensing layers for low-temperature hydrogen detection. The synthesized ZC-based material exhibited a hierarchical morphology consisting of porous microspheres and star-shaped assemblies, providing a multiscale framework for gas access and surface reactions. By varying the GO content from 0.1 to 1 wt% at a fixed Pt/C loading, the ZC-0.5G composite achieved the most balanced structure, with well-distributed GO sheets, preserved star–sphere morphology, the highest specific surface area (53.6 m^2^/g), and the largest pore volume (0.09 cm^3^/g). The optimized sensor gave responses of 12.96%, 19.20%, 22.87%, and 26.43% for 500, 4000, 8000 and 10,000 ppm H_2_ concentrations, respectively, with measurable response down to 50 ppm. The highest sensing performance was achieved at 50 °C and 60% relative humidity (RH), where the hierarchical oxide framework, GO-assisted interfacial pathways, and Pt catalytic sites acted in concert. The sensor also showed repeatable cyclic behavior and preferential response to H_2_ compared to methanol, isopropanol, ethanol, acetone, and dimethylformamide. The improved sensing performance is attributed to the synergistic combination of the hierarchical ZC framework, GO-assisted interfacial pathways, and Pt-assisted catalytic activation, which together facilitate gas diffusion, surface reactions, and resistance modulation.

## 1. Introduction

Hydrogen (H_2_) is widely considered for future sustainable energy systems due to its high gravimetric energy density and clean combustion that produces only water as a byproduct. Hydrogen is the most abundant element in the universe and is increasingly important in chemical production, oil refining, aerospace technologies, and fuel cell systems [1,2]. Despite these advantages, H_2_ poses serious safety challenges due to its wide flammability range (4–75% vol. in air), low ignition energy, and small molecular size, which allows rapid diffusion and leakage through storage and transportation systems. Because H_2_ is colorless, odorless, and tasteless, its leakage is difficult to detect and significantly increases the risk of fire and explosion. Consequently, the development of reliable, sensitive, and rapid H_2_ sensors is essential for the safe implementation of H_2_-based technologies in industrial and domestic environments [3,4,5].

Beyond safety monitoring, H_2_ sensing is also important from a materials science perspective because it provides insight into hydrogen–solid interactions at the nanoscale, which is relevant to hydrogen storage materials, catalytic interfaces, and energy conversion systems [6]. To date, various sensing materials—including noble metals, metal oxide semiconductors, carbon-based nanomaterials, and conductive polymers—have been investigated for H_2_ detection [7]. Among the various sensing platforms, metal oxide semiconductor (MOS)-based chemiresistive sensors remain the most studied due to their simple fabrication, low cost, high sensitivity, and good long-term stability [8,9,10,11]. However, achieving high response under mild operating conditions while maintaining fast kinetics, selectivity, and environmental stability remains a major challenge.

Recent advances in H_2_ chemiresistive sensors have focused on hierarchical nanostructure engineering, interface modulation, two-dimensional (2D) material integration, and noble metal sensitization to improve low-temperature sensing performance. These strategies have significantly enhanced gas diffusion, catalytic activation, and interfacial charge transfer. However, practical challenges remain, including the trade-off between low-temperature performance and fast surface reaction kinetics, limited selectivity toward hydrogen in complex atmospheres, humidity-dependent response changes, and long-term operational stability. Therefore, the development of sensing materials that simultaneously provide efficient gas transport, abundant and accessible active sites, effective interfacial charge modulation, and catalytic activation of hydrogen remains an important research goal [11,12].

ZnCo_2_O_4_ (ZC) spinel has emerged as a promising metal oxide semiconductor for gas sensing due to its stable spinel framework, mixed metal redox activity, and surface reactivity. In its cubic spinel lattice, Zn^2+^ ions mainly occupy tetrahedral sites, while Co^3+^ ions occupy octahedral sites, providing a chemically active oxide framework for oxygen adsorption and gas-induced surface redox reactions [13,14]. Therefore, in the current sensing system, ZC acts as the primary semiconductor sensing matrix, while its hierarchical porous architecture enhances H_2_ diffusion and exposes accessible reaction sites. However, pristine ZC may still exhibit limited surface accessibility, surface charge modulation, and selectivity, which requires further morphology and interface engineering [15,16]. Hybridization with graphene oxide (GO) provides oxygen-containing functional groups and defect-rich adsorption sites, suppresses oxide aggregation, and creates extensive oxide–GO interfaces that contribute to resistance modulation during gas exposure [17,18]. However, GO loading needs to be optimized because insufficient GO provides limited surface coverage, while excessive GO can block the active sites of ZC and limit gas access.

Morphological engineering is another important factor in controlling gas-sensing performance. Hierarchical architectures can improve sensor response by increasing surface accessibility, shortening gas diffusion pathways, and creating abundant surface reaction sites [19,20]. In particular, porous microspheres can facilitate gas diffusion and provide accessible adsorption domains, while star-like structures can offer exposed edges and interparticle contact points for surface reactions and resistance modulation. The coexistence of different morphologies within one material system has been observed in several self-assembled and hydrothermally prepared materials, where synthesis conditions direct parallel growth or self-organization pathways [21,22].

In addition to the morphology and interface engineering, the Pt nanoparticles provide the catalytic sensitization of the ZC-G sensor surface. Pt enhances the dissociation of H_2_ molecules into active hydrogen species, which subsequently migrate to the oxide surface via a spillover pathway and react with adsorbed oxygen species. This process accelerates the surface reaction kinetics at relatively low operating temperatures. Pt can also contribute to the electronic sensitization by modulating the local surface barrier and charge transfer behavior at the Pt/ZC contact. As a result, the complementary functions of the hierarchical ZC sensor matrix, the GO-mediated surface and interfacial adsorption network, and the Pt-assisted catalytic activation collectively enhance the gas accessibility, surface reaction kinetics, and resistance modulation during H_2_ exposure. Pt-assisted H_2_ dissociation, spillover, and metal-semiconductor contact modulation are well-established mechanisms in Pt-decorated metal oxide H_2_ sensors [23,24].

Previous studies have demonstrated the potential of spinel-type metal oxides, ZC-based materials, and GO-modified metal oxide systems for gas sensing. Spinel ferrite/oxide semiconductors have been widely investigated as resistive gas-sensing materials, where exposure to reducing gases can modulate the sensor resistance through surface reactions with adsorbed oxygen species [25]. ZC-based nanostructures have also been explored for detecting gases such as liquefied petroleum gas, formaldehyde, triethylamine, and H_2_S, confirming the relevance of this spinel oxide family for gas-sensing applications. In parallel, GO-modified metal oxide sensors have shown particular promise for H_2_ sensing; for example, Rasch et al. reported a GO-covered ZnO microwire sensor in which the GO membrane acted as a molecular-sieving layer, enabling selective room-temperature H_2_ detection [26]. These reports suggest that combining a semiconducting oxide framework with GO-based interfacial/selective transport effects can be useful for gas sensing. However, studies combining hierarchical ZC star–sphere architectures, optimized GO loading, and Pt-assisted catalytic sensitization for H_2_ sensing remain limited.

In this work, hierarchical ZC star–sphere architectures integrated with GO and Pt nanoparticles were synthesized and systematically investigated for H_2_ sensing. The design goal is based on the simultaneous optimization of three key factors: dual hierarchical morphology for gas diffusion and active site exposure, GO-mediated oxide–carbon interfaces for charge transfer, and Pt-assisted catalytic activation for hydrogen dissociation. By tuning the GO content from 0.1 to 1 wt%, the relationship between the composite architecture and sensing performance was clarified. The optimized ZC-0.5G sensor showed improved H_2_ response under mild operating conditions, and the best performance was achieved at 50 °C and 60% relative humidity (RH). The results demonstrate that coupling the star–sphere morphology of ZC with GO surface networks and Pt catalytic sites provides an effective strategy for developing high-performance metal oxide/carbon hybrid sensors for H_2_ detection.

## 2. Experimental

### 2.1. Materials

Zinc nitrate hexahydrate (Zn(NO_3_)_2_·6H_2_O, Merck (Darmstadt, Germany), ≥99%), cobalt nitrate hexahydrate (Co(NO_3_)_2_·6H_2_O, Merck, ≥99%), urea (Sigma-Aldrich, St. Louis, MO, USA, ≥99%), ammonium fluoride (NH_4_F, Sigma-Aldrich, ≥98%), ethylene glycol (Merck, ≥99.5%), ethanol (EtOH, Merck, ≥99.8%), a commercial platinum catalyst supported on graphitized carbon (20 wt% Pt, Pt/C; Sigma-Aldrich), isopropanol (IPA, Sigma-Aldrich, ≥99.7%), polyvinylpyrrolidone (PVP, Sigma-Aldrich), and Nafion solution (5 wt%, Sigma-Aldrich) were used as received. Graphite powder (Sigma-Aldrich, ≥99.5%), concentrated sulfuric acid (H_2_SO_4_, 98%, Sigma-Aldrich), potassium permanganate (KMnO_4_, Merck, ≥99%), hydrogen peroxide (H_2_O_2_, 30%, Merck), and hydrochloric acid (HCl, 37%, Sigma-Aldrich) were used for the synthesis of GO by the modified Hummers method. Deionized (DI) water was used throughout all synthesis and washing steps.

### 2.2. Synthesis of ZC

The ZC precursor was prepared by dissolving 1.868 g of Zn(NO_3_)_2_·6H_2_O and 3.655 g of Co(NO_3_)_2_·6H_2_O in 60 mL of DI water under continuous stirring for 20 min. Subsequently, 20 mL of ethylene glycol was added dropwise to enhance solvation and control the nucleation environment [27], and the mixture was stirred for an additional 10 min. A urea solution (0.6 g in 5 mL DI water) was then added as a slow-release source of OH^−^, followed by the incorporation of NH_4_F (0.15 g), which acts as a facet-directing agent; the suspension was further stirred for 10 min to ensure homogeneous mixing. The final mixture was transferred into a Teflon-lined autoclave and subjected to hydrothermal treatment at 180 °C for 12 h. After cooling naturally to room temperature, the resulting solid was collected by centrifugation and thoroughly washed with DI water and EtOH. The product was dried at 70 °C for 12 h and subsequently calcined at 400 °C for 2 h to obtain the crystalline ZC spinel.

### 2.3. Synthesis of GO

GO was synthesized via a modified Hummers method [28]. Briefly, 2 g of graphite was dispersed in 60 mL of concentrated H_2_SO_4_ under ice-bath conditions to ensure controlled oxidation, and the mixture was stirred for 30 min. KMnO_4_ (6 g) was then added slowly to prevent excessive heat evolution, and oxidation was allowed to proceed for an additional 30 min. The reaction temperature was subsequently raised to 40 °C and maintained for 2 h to promote layer expansion and oxygen functionalization. Afterward, 250 mL of DI water was introduced dropwise, followed by continuous stirring for 30 min to dilute the reaction medium. The oxidation process was terminated by the gradual addition of 10 mL of 30% H_2_O_2_, which yielded a characteristic bright-yellow suspension. The resulting product was purified by repeated centrifugation and washing with 5% HCl and DI water to remove manganese species and acid residues [29]. Finally, the GO was dried under vacuum at 60 °C for 24 h to obtain well-exfoliated, oxidation-rich GO.

### 2.4. Preparation of Pt/C-Decorated Composites with Different GO Loadings

Pt/C-decorated ZC–G composites with nominal GO contents of 0.1, 0.5, and 1 wt% relative to ZC were prepared using a dispersion–coupling route. For each composition, 0.330 g of ZC powder was dispersed in 10 mL of a 1:1 (*v*/*v*) DI/EtOH mixture and ultrasonicated for 10 min. GO amounts of 0.33, 1.65, and 3.30 mg were separately dispersed in 5 mL of the same solvent mixture to prepare the ZC-0.1G, ZC-0.5G, and ZC-1G composites, respectively. Each GO dispersion was added slowly to the corresponding ZC suspension under continuous stirring, followed by 10 min of ultrasonication.

**Figure 1:**
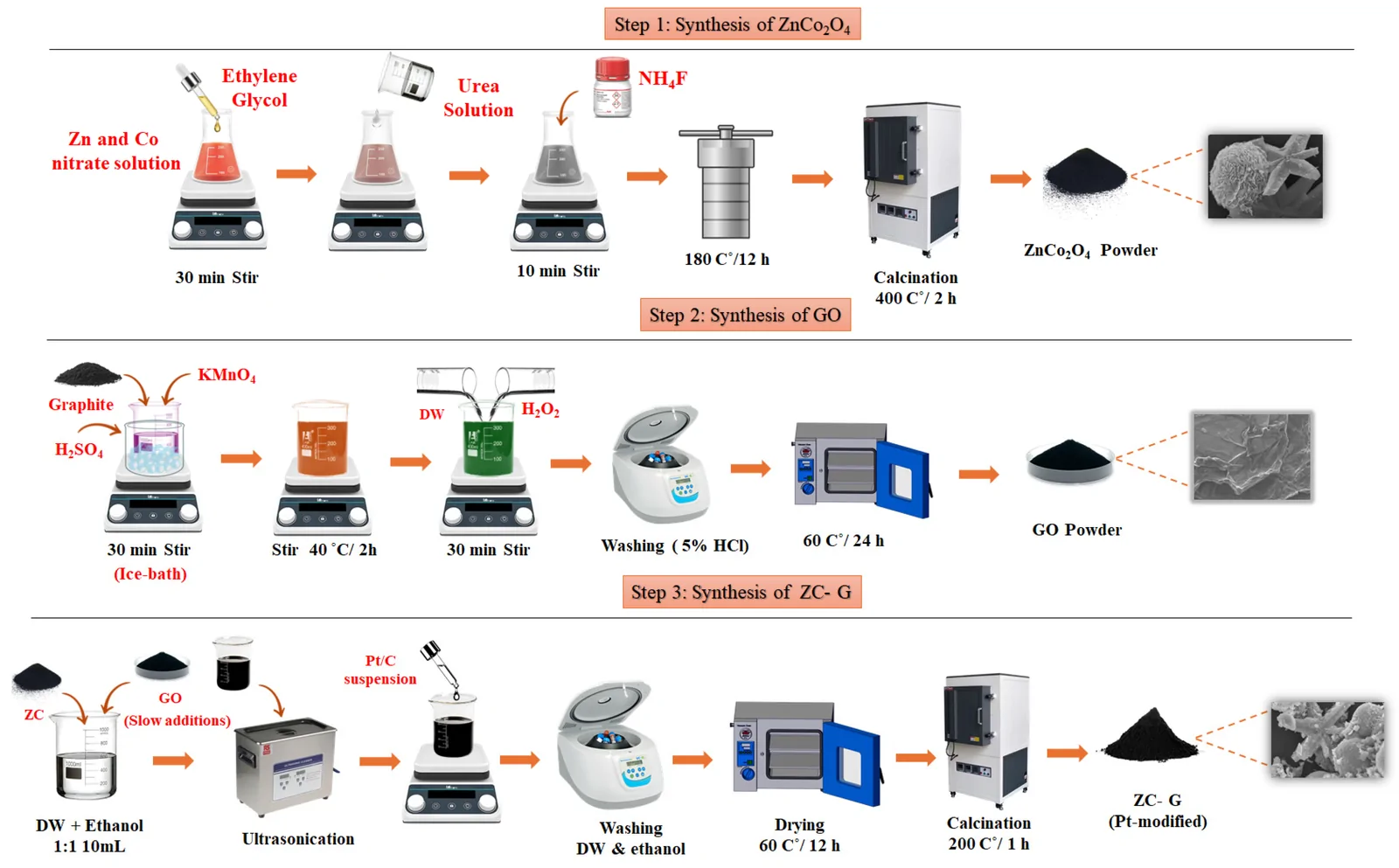
Schematic illustration of the synthesis routes: Step 1, hydrothermal synthesis of hierarchical ZC; Step 2, synthesis of GO by the modified Hummers method; and Step 3, incorporation of GO and a fixed Pt/C loading into ZC to obtain the Pt/C-decorated ZC–G composites.

A commercial catalyst containing 20 wt% Pt supported on graphitized carbon was used as the Pt source. For each batch, 9 mg of Pt/C, containing 1.8 mg of elemental Pt and corresponding to an approximate nominal Pt loading of 0.5 wt% with respect to the total composite mass, was dispersed in 5 mL of a 1:1 (*v*/*v*) DI water/EtOH mixture. The resulting Pt/C suspension had a concentration of 1.8 mg Pt/C mL^−1^ and was ultrasonicated until homogeneous. It was then added dropwise to the ZC–GO suspension under continuous stirring, followed by an additional 10 min of ultrasonication. The same Pt/C amount and incorporation procedure were used for all composites. The suspensions were stirred for 30 min, washed repeatedly with DI water and EtOH, and dried at 60 °C for 12 h. Finally, the powders were thermally treated at 200 °C for 1 h. The resulting composites were denoted as ZC-0.1G, ZC-0.5G, and ZC-1G. A schematic illustration of the synthesis routes for ZC, GO, and the ZC–G composites is shown in Figure 1.

For the control experiments, ZC–GO containing 0.5 wt% GO was prepared without Pt/C, whereas ZC–Pt was prepared using the same Pt/C loading without GO. All other processing conditions were kept unchanged.

### 2.5. Fabrication of Sensing Layers and Gas-Sensing Measurements

**Figure 2:**
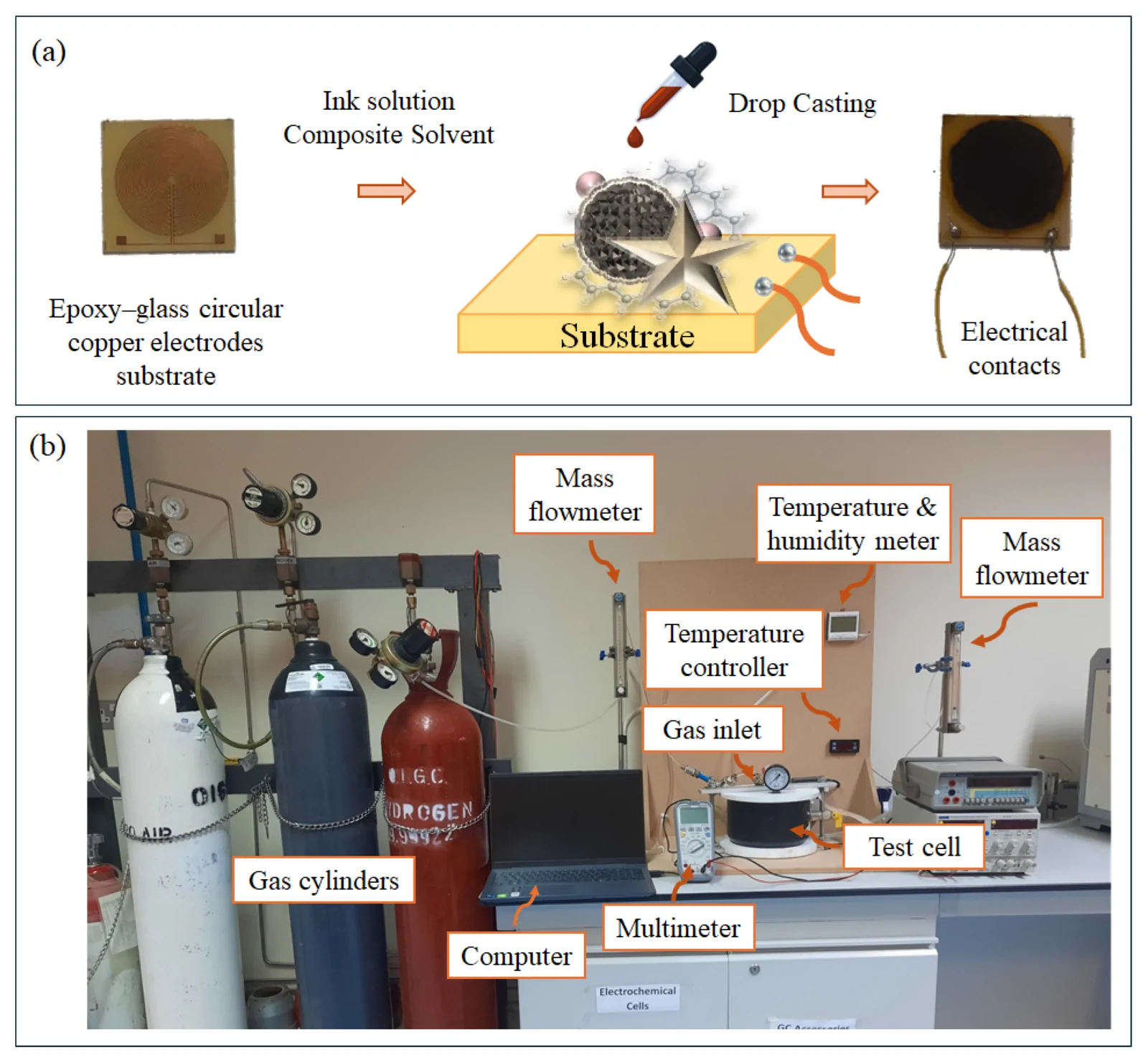
(**a**) Drop-casting fabrication of the sensing layer on an epoxy–glass substrate containing circular copper electrodes and (**b**) photograph of the gas-sensing measurement setup.

The sensing layers were fabricated using the synthesized composites by the drop-casting method, as illustrated in Figure 2a. In a typical procedure, 5 mg of composite powder was dispersed in a mixture of 800 μL EtOH and 200 μL DI water and sonicated for 20 min to obtain a homogeneous dispersion. Subsequently, 0.5 mg of PVP and 10 μL of Nafion solution (5 wt%) were added, where PVP served as a dispersing agent to improve particle dispersion and minimize agglomeration, while Nafion acted as a film-forming binder to enhance film adhesion and mechanical stability on the substrate. These additives were employed primarily to improve particle dispersion, coating uniformity, film adhesion, mechanical stability, and fabrication reproducibility. The resulting mixture was further sonicated for 10 min to obtain a stable and homogeneous sensor ink suitable for coating.

Circular copper electrodes fabricated on 3 × 3 cm^2^ glass–epoxy substrates were thoroughly cleaned by successive washing with DI water, EtOH, and acetone to remove surface contaminants and improve coating adhesion. For each sensor, the prepared sensing ink was deposited onto the electrode area in three successive drop-casting steps (~0.3–0.4 mL per deposition). After each deposition step, the coated substrate was dried at 60 °C for 10 min to promote solvent evaporation and improve film uniformity. The coated sensors were then dried under vacuum for 1 h to remove residual solvent. Finally, the sensing layers were thermally treated at 100 °C for 1 h in air to improve coating adhesion and stabilize the sensor layer before gas-sensing measurements. Gas sensing measurements were performed in a sealed chamber with a total volume of 2 L. The desired H_2_ concentrations were obtained by independently controlling the flow rates of H_2_ (99.99%) and N_2_ flow rates using separate calibrated flowmeters, with both gas streams introduced into the sensing chamber at the prescribed flow rates. Sensing measurements were carried out at operating temperatures of 40–60 °C and RH levels of 40–60%. The ambient laboratory humidity (approximately 60% RH) was used as the reference condition. Lower RH levels (40% and 50%) were achieved by placing different amounts of molecular sieve desiccant inside the sealed sensing chamber. Before each measurement, the chamber was flushed with nitrogen and allowed to equilibrate at the selected temperature and RH until a stable baseline resistance was achieved.

The electrical resistance of the sensor was monitored during the sensing test using a digital multimeter. The H_2_ sensing response was calculated from the relative resistance change as follows:

(1) $$\mathrm{Response}\;=\;\frac{R_{a}-\;R_{g}}{R_{a}}\;\times 100$$

where $R_{a}$ is the stable baseline resistance under the nitrogen atmosphere and $R_{g}$ is the resistance measured during H_2_ exposure. The response time ($t_{res}$) was defined as the time required to reach 90% of the total resistance change after H_2_ introduction, whereas the recovery time ($t_{rec}$) was defined as the time required to recover 90% of the resistance change after H_2_ removal and nitrogen purging. A schematic illustration of the experimental sensor setup is presented in Figure 2b.

### 2.6. Characterization Techniques

The phase composition and crystallographic structure of the synthesized materials were investigated by X-ray diffraction (XRD) using a Rigaku Miniflex diffractometer (Rigaku Corporation, Tokyo, Japan) equipped with Cu Kα radiation (λ = 1.5406 Å). Diffraction patterns were recorded over the 2θ range of 10–80°. The morphological features, elemental composition, and spatial distribution of elements were examined using field-emission scanning electron microscopy (FESEM, JEOL JSM-7600F, JEOL Ltd., Tokyo, Japan) operated at an accelerating voltage of 15 kV. Nanoscale structural information was obtained using transmission electron microscopy (TEM) and high-resolution transmission electron microscopy (HRTEM) with a JEOL JEM-2100F microscope (JEOL Ltd., Tokyo, Japan) operated at an accelerating voltage of 200 kV. Fourier transform infrared (FTIR) spectra were recorded using a Spectrum BX spectrometer (PerkinElmer Inc., Waltham, MA, USA) over the range of 400–4000 cm^−1^. Optical absorption properties were evaluated using ultraviolet–visible (UV–Vis) diffuse reflectance spectroscopy with a Lambda 25 spectrometer (PerkinElmer Inc., Waltham, MA, USA). The specific surface area and textural properties were investigated by nitrogen adsorption–desorption measurements at 77 K using a Micromeritics surface area analyzer (Micromeritics Instrument Corporation, Norcross, GA, USA). The specific surface area was calculated using the Brunauer–Emmett–Teller (BET) method, while the pore size distribution was determined from the desorption branch using the Barrett–Joyner–Halenda (BJH) method. The surface elemental composition and chemical states of the optimized ZC-0.5G composite were investigated by X-ray photoelectron spectroscopy (XPS) using a Scienta Omicron instrument (Scienta Omicron GmbH, Taunusstein, Germany). Spectral fitting was performed using CasaXPS software (version 2.3.26PR1.0; Casa Software Ltd., Teignmouth, Devon, UK).

## 3. Results and Discussion

### 3.1. Structural and Phase Analysis by X-Ray Diffraction

**Figure 3:**
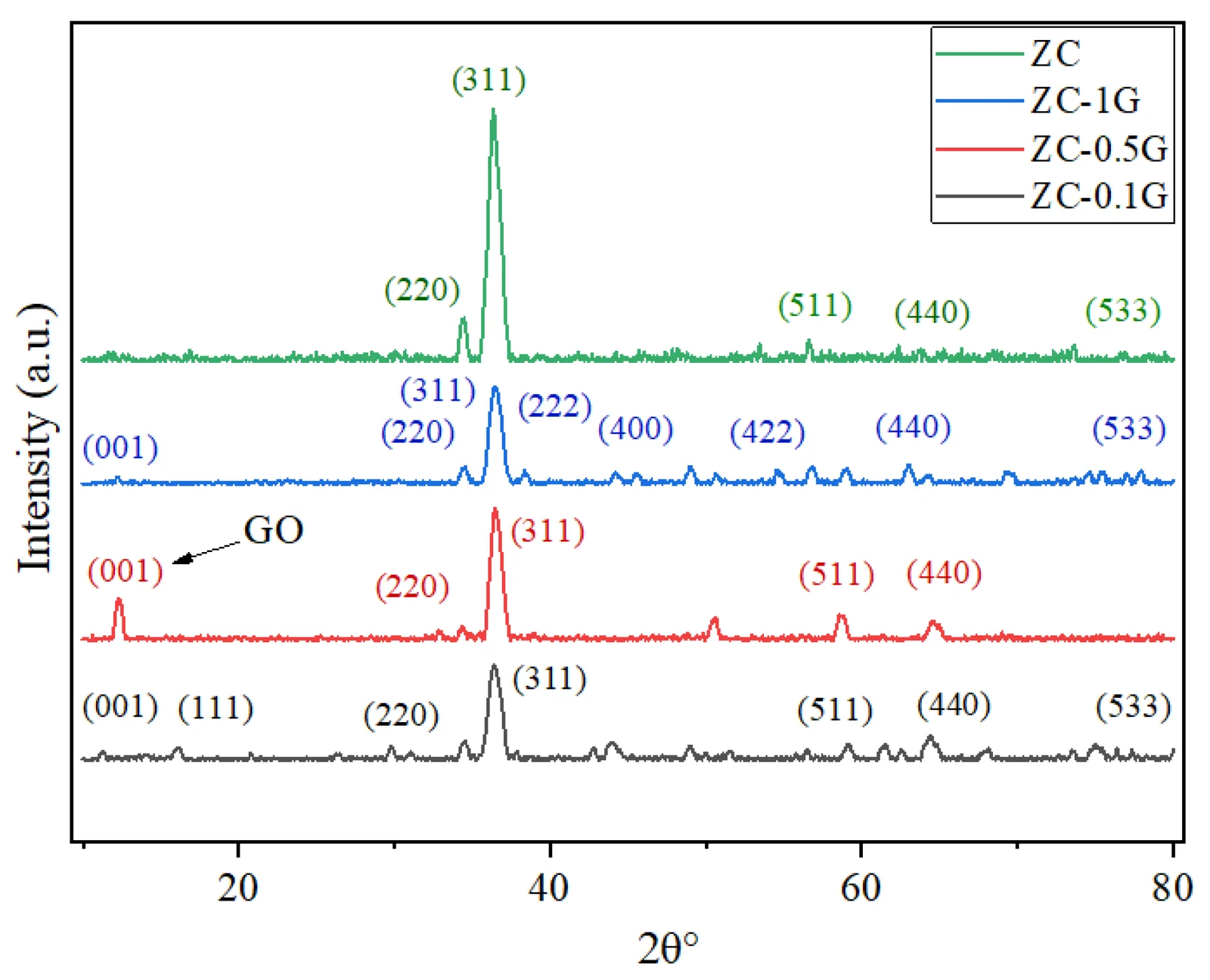
XRD patterns of ZC and ZC-G composites with different GO contents (0.1, 0.5, and 1 wt%) recorded in the 2θ range of 10–80°.

The crystal structure of the synthesized samples was investigated by XRD in the 2θ range from 10 to 80° (Figure 3). The ZC diffraction pattern shows well-defined peaks characteristic of the cubic spinel phase of ZC with the space group Fd-3m and is in good agreement with the standard reference pattern ICDD/PDF No. 23-1390. The diffraction peaks observed at approximately 18.9°, 31.2°, 36.8°, 38.5°, 44.8°, 55.6°, 59.3°, 65.2° and 77° are attributed to the crystallographic planes (111), (220), (311), (222), (400), (422), (511), (440) and (533) of ZC spinel, respectively [30,31]. The most intense diffraction peak in pristine ZC and all ZC–G composites is located at approximately 36.8° and is indexed to the (311) crystallographic plane. Although the relative intensities of the weaker reflections, including the (222) peak, vary slightly among the composites, the (311) reflection remains the dominant diffraction peak in all samples. Other characteristic peaks of the spinel phase appear at almost the same angles in the original ZC and composite samples, indicating that the incorporation of GO and Pt/C does not change the crystallographic structure of the ZC lattice [32]. The XRD patterns of the ZC-G composites are very similar to those of the reference ZC sample, with differences mainly observed in peak intensity and broadening. Such changes are commonly reported for composites and are associated with changes in crystallite size and lattice coherence caused by interfacial interactions with carbon phases, without the appearance of additional crystalline oxide phases [33]. A weak diffraction feature observed just above 10° in the composite samples is related to the (001) reflection of GO, which arises from the enlarged interlayer spacing caused by the oxygen-containing functional groups. Due to the low GO content (0.1–1 wt%) and the dominant diffraction intensity of the ZC crystalline phase, this reflection appears with low intensity and does not affect the overall diffraction structure of the composites [29,34,35,36].

### 3.2. Morphological Characteristics of Dual Hierarchical Star–Sphere ZC Architectures

**Figure 4:**
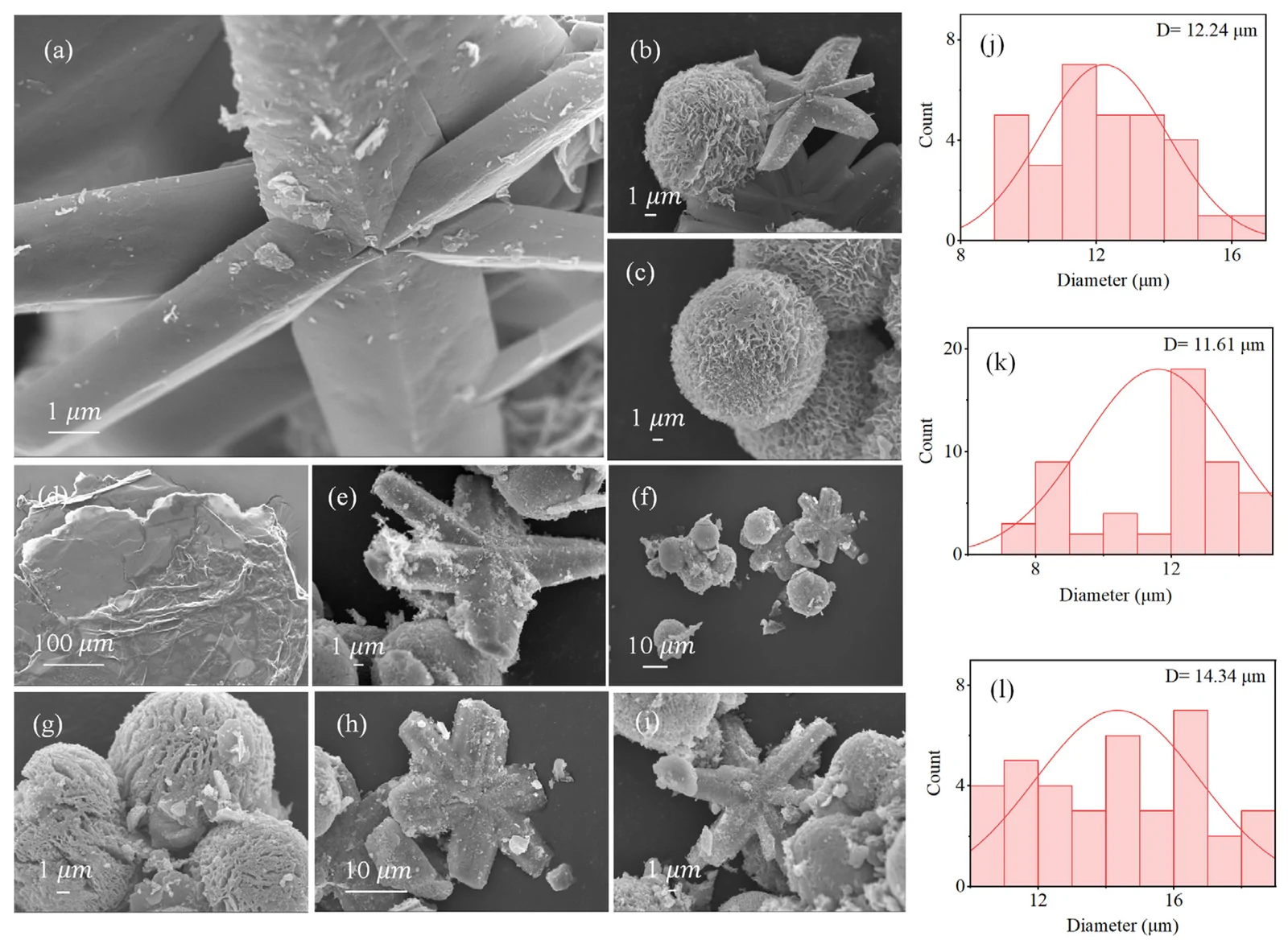
(**a**–**c**) FESEM micrographs of pristine ZC microspheres and star-like architectures, (**d**) GO, and the ZC-G nanocomposites with 0.1 wt% (**e**), 0.5 wt% (**f**–**h**), and 1 wt% GO (**i**). (**j**–**l**) Microsphere diameter distributions of the ZC-0.1G, ZC-0.5G, and ZC-1G composites, respectively, obtained from FESEM images using ImageJ software (National Institutes of Health, Bethesda, MD, USA). The solid curves represent Gaussian fits to the measured size distributions.

FESEM images reveal the specific morphological features of each component in the ZC-G system. Pure ZC exhibits two distinct hierarchical architectures, including micro stars composed of radially oriented nanorods (Figure 4a) and porous microspheres (Figure 4b). The coexistence of these two morphologies (Figure 4c) can be related to the hydrolysis of urea, which gradually supplies ${OH}^{-}$ ions and regulates precursor precipitation and self-assembly into microspheres, while NH_4_F acts as a morphology-directing agent by modifying crystal growth and promoting anisotropic star-like architectures [21,37,38]. The highly porous shell of the microspheres indicates the aggregation of primary nanocrystals. Figure 4d for GO shows folded and stacked nanosheets with typical layered folding produced under mild oxidation conditions [39,40]. In the composite containing 0.1 wt% GO (Figure 4e), GO nanosheets appear associated with the oxide surface without causing significant changes in the intrinsic morphologies of ZC. These sheets appear to be partially interspersed throughout the microspheres and star-shaped units, suggesting a high affinity of GO functional groups for spinel surfaces. The retention of both morphologies suggests that GO acts primarily as a surface modifier rather than a growth directing agent at this level [41]. For ZC-0.5G (Figure 4f–h), the GO sheets appear more uniformly distributed over the preformed ZC structures, providing extended oxide–carbon interfacial contact while preserving the hierarchical dual star–sphere morphology. The moderate GO loading effectively limits secondary particle aggregation and maintains an open porous architecture, thereby increasing the accessibility of the oxide surface and facilitating gas diffusion through the sensing layer [42]. The coexistence of well-defined spheres and star-like clusters is consistent with the elemental mapping, which indicates the presence of zinc, cobalt, oxygen, carbon and platinum-containing regions across the composite. The coexistence of porous microspheres and star-like structures is expected to provide complementary functions. The microspheres offer a porous framework for gas diffusion and adsorption, while the star-like structures expose high-energy edges and facets that facilitate surface reactions and charge transfer [43,44].

Morphology optimization in the present work was achieved by systematically adjusting the GO loading while maintaining the same hydrothermal synthesis conditions and Pt content for all composites. The ZC-0.5G sample showed the most homogeneous distribution of GO sheets without disturbing the hierarchical ZC star–sphere architecture. At this optimized loading, GO effectively suppressed particle aggregation, maintained open diffusion pathways, and promoted intimate oxide–GO interfacial contact, which is consistent with the highest specific surface area (53.6 m^2^/g) and pore volume (0.09 cm^3^/g). In contrast, insufficient GO (0.1 wt%) provided limited interfacial coverage, while excessive GO (1 wt%) partially covered the star-like structures and reduced surface accessibility. These morphology-dependent structural changes contribute to the superior H_2_ sensing performance of the optimized ZC-0.5G composite [45,46]. At the highest GO loading (1 wt%; Figure 4i), the star-like units remain visible but appear partially covered and less well defined, which may be associated with greater surface coverage and secondary aggregation or restacking of GO [41,47]. This results in broader and less defined edges and a partial loss of the characteristic radial geometry of the initial stars.

Figure 4j–l presents the microsphere diameter distributions obtained from FESEM images of ZC-0.1G, ZC-0.5G, and ZC-1G, respectively, using ImageJ analysis. The three composites exhibit unimodal, near-Gaussian distributions, with average diameters of approximately 12.24, 11.61, and 14.34 μm, respectively. Among the investigated composites, ZC-0.5G shows the smallest average microsphere diameter and the most compact distribution, consistent with the relatively uniform dispersion observed in the corresponding FESEM images. The increase in apparent diameter for ZC-1G may be associated with greater GO coverage and secondary aggregation at the higher loading. These GO-loading-dependent differences in particle organization can influence surface accessibility and gas diffusion pathways within the sensing layer [48,49].

### 3.3. Nanoscale Structural Analysis by Transmission Electron Microscopy

**Figure 5:**
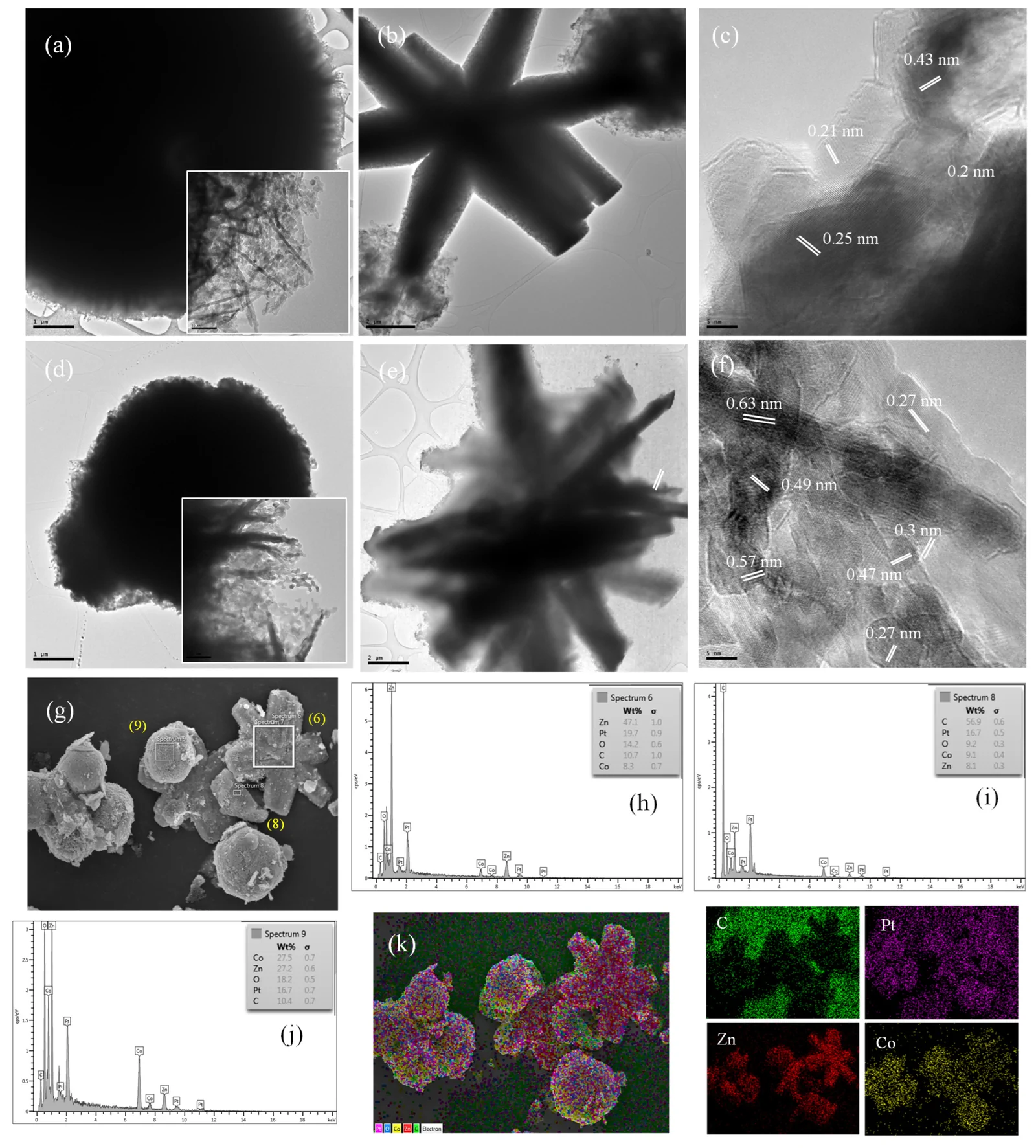
TEM images of (**a**,**b**) ZC and (**d**,**e**) ZC-0.5G. HRTEM of (**c**,**f**) ZC and ZC- 0.5G, respectively. (**g**) FESEM image of the ZC-G nanocomposite showing selected regions for point-EDS analysis. (**h**–**j**) EDS spectra from the marked points. (**k**) Elemental mapping of the studied area, along with separate maps showing the spatial distribution of C, Pt, O, Co, and Zn in the spherical and star-shaped structures of the composite.

Transmission electron microscopy (TEM) was used to investigate the nanoscale architecture of the ZC-0.5G composite and the spatial association between ZC nanocrystallites and GO-derived sheets. As shown in Figure 5a,b, ZC exhibits two simultaneous morphologies–porous microspheres and star-like architectures formed by sheet/plate-shaped subunits. This resulting structure is characteristic of hierarchical ZC spinel grown under competitive kinetic nucleation/growth conditions, where anisotropic attachment and directional aggregation can create branched stars alongside spherical aggregates. This dual morphology, reported for spinel cobaltites, could be attractive for sensing because porosity, as a gas diffusion facilitator, is combined with abundant edge/defect-rich surfaces to increase available adsorption/reaction sites [44]. HRTEM images of the pristine ZC (Figure 5c) show lattice fringes of approximately 0.43, 0.25, and 0.20–0.21 nm. These values are reasonably associated with the (111), (311), and (400) planes of cubic ZC, respectively, consistent with the XRD results.

After hybridization, TEM images of the ZC-0.5G composite (Figure 5d,e) show thin, transparent, and wrinkled sheets on and among the ZC units, consistent with GO-derived layers. The two-dimensional GO sheets provide extended contact surfaces around the preformed ZC particles, which can improve their spatial dispersion, reduce secondary aggregation, and increase oxide–carbon interfacial contact relevant to charge transfer in resistive sensing [50]. Inside the composite, the star-like ZC clusters remain clearly recognizable, together with the rough contrast of the porous microspheres, indicating that the post-synthetic incorporation of GO preserves the hierarchical ZC framework while introducing GO-derived sheets around and between the preformed ZC structures [50]. In these integrated structures, the GO sheets provide an extended interfacial support network and additional charge-transport junctions, while the spinel provides the primary chemisorption/catalytic surface for gas reactions [51]. HRTEM of the ZC-0.5G composite (Figure 5f) shows multiple lattice spacings. The spacings of approximately 0.27–0.30 nm are primarily consistent with the (220) reflection of ZC, whereas the larger spacings of approximately 0.47–0.63 nm are attributed to locally expanded and heterogeneous interlayer regions of the GO-derived sheets [52]. The presence of these lattice spacings in a region is a direct result of oxide–carbon coupling. Notably, the oxide lattice fringes remain continuous up to the oxide–graphene interface, indicating close interfacial adhesion. Such close connections, in addition to maintaining abundant catalytic/chemisorption sites on the oxide surface, facilitate efficient charge transfer between the ZC semiconductor and the graphene-derived interfacial network [53]. Pt nanoparticles at low loadings are not clearly separated into discrete crystalline domains in TEM images [54,55]. Therefore, the presence and chemical state of Pt are better supported by EDS mapping and XPS. Overall, TEM analysis confirms that the optimized ZC-0.5G composite retains the hierarchical spinel morphology of ZC, while integrating graphene-derived sheets as an interfacial support network. The simultaneous presence of porous spheres, star-shaped architectures, and graphene layers, along with well-resolved lattice fringes of both components, creates a robust nanoscale framework that promotes efficient gas diffusion, rich active sites, and enhanced interfacial charge transport.

### 3.4. Elemental Composition and Spatial Distribution

EDS analysis of the ZC-0.5G nanocomposite reveals two distinct but compositionally related morphologies—porous microspheres and star-shaped structures—each with different elemental distributions while maintaining the Zn-Co-O-rich regions across the composite (Figure 5g). EDS of a spot taken from the star-shaped region (Figure 5h) reveals Zn and Co as the main metallic components, along with moderate Pt and a minor carbon signal, indicating a Zn-rich oxide domain with detectable Co and Pt-containing species. In contrast, the porous sphere (Figure 5i) exhibits significantly higher carbon and Pt contents along with lower Zn/Co intensities, indicating a carbon- and Pt-enriched region near the microsphere surface. The third region (Figure 5j) shows stronger Zn, Co, and O signals with moderate Pt intensity, indicating a more oxide-rich Zn–Co–O region within the composite. This element distribution pattern is consistent with the literature on ZC-G hybrid structures, where carbon sheets and noble metals are preferentially adsorbed on high-energy surfaces and in interparticle junctions [56]. Elemental mapping in Figure 5k supports this interpretation. The Zn and Co maps show a uniform spatial overlap in both morphologies, consistent with the presence of the Zn–Co–O framework across both morphological domains. The O map follows the spatial distributions of Zn and Co, showing extensive overlap among Zn, Co, and O across both morphological domains. In contrast, the C and Pt maps show greater signal intensity around the outer regions of the microspheres, indicating surface enrichment of the carbon-supported Pt component [57,58,59,60].

### 3.5. FTIR Analysis

**Figure 6:**
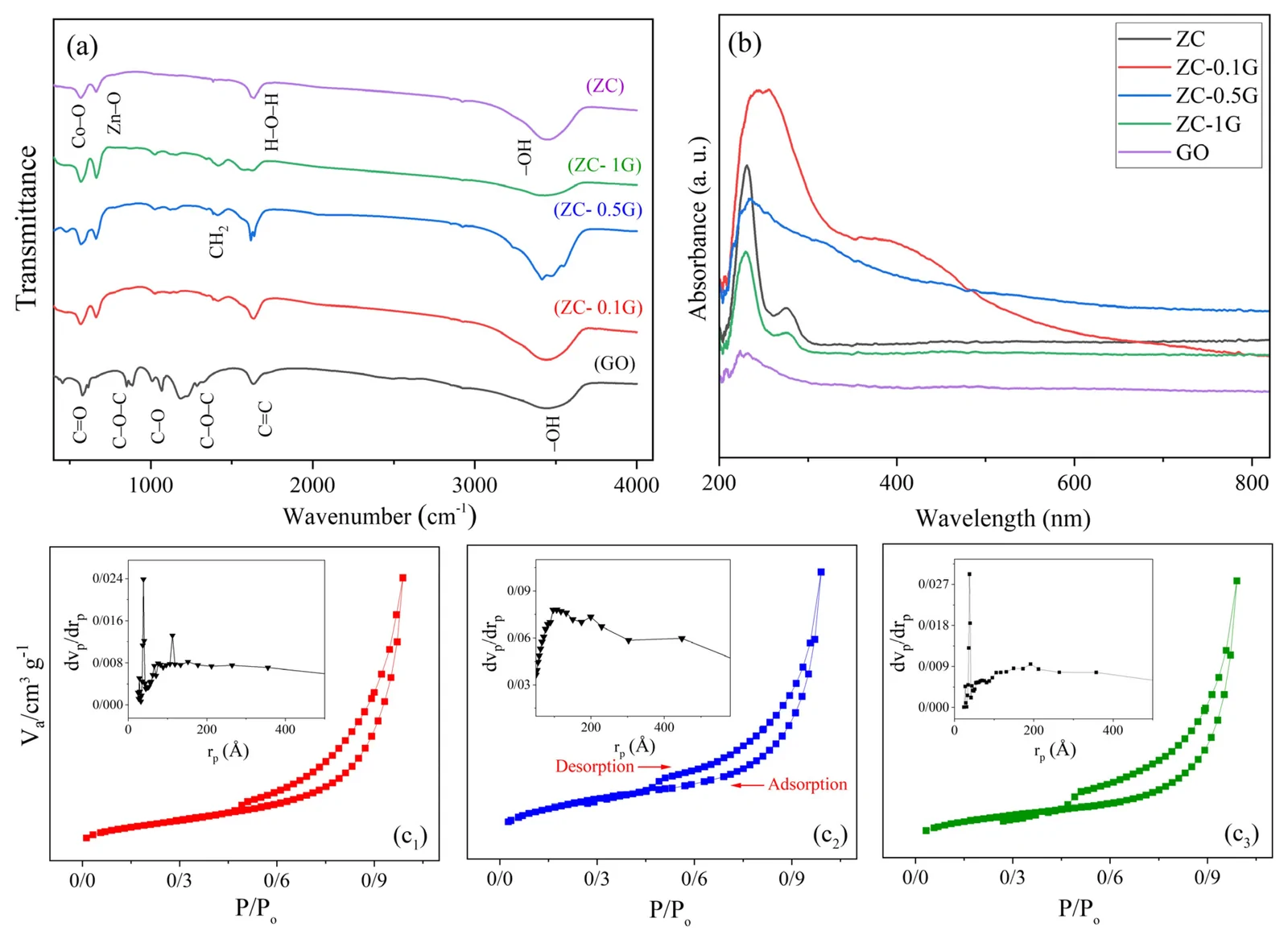
(**a**) FTIR and (**b**) UV-Vis spectra of pristine GO, pure ZC, and ZC–(0.1–1)G nanocomposites. N_2_ adsorption−desorption isotherm plots of (**c1**) ZC-0.1G; (**c2**) ZC-0.5G; (**c3**) ZC-1G; Inset represents the pore size distribution results calculated from the BJH desorption pore volume data for the respective sample.

Figure 6a shows the FTIR spectra of GO, ZC spinel, and ZC-G nanocomposites. The pristine GO exhibits a broad O-H stretching band at ~3440–3450 cm^−1^, along with a band at ~1620–1635 cm^−1^, which is usually attributed to C=C vibrations with a δ(H–O–H) contribution from adsorbed water. Strong C–O and C–O–C stretching vibrations are observed in the region of 1000–1250 cm^−1^, while the C=O band near ~1720 cm^−1^ appears weak, an FTIR feature often reported for GO synthesized via modified Hummers methods depending on the degree of oxidation and post-synthesis conditions [61]. Weak absorptions below 900 cm^−1^ have been identified in the spectrum of GO and can be attributed to C–O vibrations, which are characteristic of GO sheets [62]. Pure ZC exhibits two characteristic metal-oxygen (M–O) stretching modes of the spinel structure, a band with a higher wavenumber around 660–670 cm^−1^ (ν_1_, associated with tetrahedral sites) and a band with a lower wavenumber around 560–580 cm^−1^ (ν_2_, associated with octahedral sites), supporting the formation of a ZC spinel network. Such ν_1_/ν_2_ vibrational features have been well reported for ZC and the related cobaltite spinel [63]. All ZC-G composites retain the characteristic spinel bands ν_1_ (~663 cm^−1^) and ν_2_ (~563 cm^−1^), indicating that the ZC framework is structurally preserved after the incorporation of GO and Pt/C. With increasing GO content, the relative intensity of the C–O/C–O–C region (~1020–1130 cm^−1^) becomes more prominent, consistent with the increasing contribution of oxygen-containing functional groups from GO to the composite spectra [63,64,65]. The O-H stretching band at ~3440 cm^−1^ shows a lower intensity in the composite samples compared to the pristine GO, which is attributed to the reduced contribution of free hydroxyl groups and adsorbed water after the composite formation. A slight broadening of the band at ~1630 cm^−1^ is also observed after the addition of GO, a behavior that is associated in metal oxide–GO nanocomposites with interfacial interactions between the metal oxide and graphene-based sheets [66,67]. The persistence of the band at ~663 cm^−1^ in all composite samples, which is absent in pristine GO, serves as a clear vibrational fingerprint of the ZC spinel phase [68].

### 3.6. UV–Vis Optical Properties

The UV-Vis absorption spectra of GO, ZC and ZC-G composites were recorded over the wavelength range of 200–800 nm, as shown in Figure 6b. GO exhibits an absorption band at approximately 230–240 nm, assigned to the π → π* transition of C=C bonds in sp^2^ domains, while the weak and poorly resolved contribution near 300 nm is associated with n → π* transitions of oxygen-containing groups [69,70,71]. Pristine ZC shows strong absorption in the deep-UV region between 230 and 280 nm, followed by a broad absorption tail extending into the visible range. These features are commonly associated with O^2−^ → Co^3+^ charge-transfer transitions and Co^2+^/Co^3+^ d–d transitions in the spinel structure [72].

Although the principal ZC-related absorption features are retained in all composites, their intensity, broadening, and degree of spectral overlap vary with GO loading. ZC-0.1G displays the highest overall absorbance and a broad two-feature profile, consisting of an intense UV band and a broad shoulder extending through approximately 300–450 nm. This behavior may arise from the superposition of the intrinsic ZC absorption, GO-related electronic transitions, and interfacial interactions between ZC and well-dispersed GO sheets. In ZC-0.5G, the absorption features become broader and less resolved, while a relatively extended absorption tail is maintained toward the visible region, indicating stronger overlap of the ZC- and GO-related contributions at the intermediate GO loading. By contrast, ZC-1G exhibits a spectral profile closer to that of pristine ZC and lower absorbance over part of the investigated range. This decrease may be associated with partial GO restacking or aggregation at the higher loading, which can reduce the effective interaction between incident light and the ZC particles by blocking or scattering the incident light [73,74]. Therefore, the spectra are mainly similar in the positions of the characteristic ZC absorption features,, whereas their intensity and line shape remain dependent on the GO content [75].

### 3.7. BET Surface Area and Pore Structure

The N_2_ adsorption–desorption isotherms of ZC-G composites exhibit type IV isotherms with H3/H4-type hysteresis characteristics, indicating the coexistence of gap-like pores originating from GO sheets and intrinsic interparticle porosity in spinel microspheres (Figure 6(c1–c3)). Such hybrid porosity is known to facilitate fast gas diffusion kinetics and adsorption-related surface reactions in chemiresistive sensors [76,77]. According to Table 1, among the three composites, ZC-0.5G exhibits the highest S_BET_ (53.6 m^2^/g), which is significantly larger than ZC-0.1G (26 m^2^/g) and ZC-1G (38 m^2^/g). The increase in specific surface area in ZC-0.5G is attributed to the optimized interfacial interaction between ZC microspheres and GO layers, where GO sheets prevent particle aggregation and enhance the formation of mesopores [41]. The total pore volume also follows the same trend, with ZC-0.5G showing a significant increase to 0.09 cm^3^/g compared to 0.01 cm^3^/g in both ZC-0.1G and ZC-1G. This increase in pore volume indicates that the addition of GO at a concentration of 0.5 wt% creates interconnected diffusion channels and increases mesoporosity—an effect that is known to accelerate molecular transport and improve surface reaction dynamics in gas sensing [78]. Despite the variation in surface area and pore volume, all three composites exhibit comparable average pore diameters in the range of 12–14.5 nm. Such structural features can increase active site exposure, reduce diffusion resistance, and improve charge transport pathways as key factors in controlling the amplitude and kinetics of the sensor response [79].

### 3.8. Surface Chemical Composition and Oxidation States

**Figure 7:**
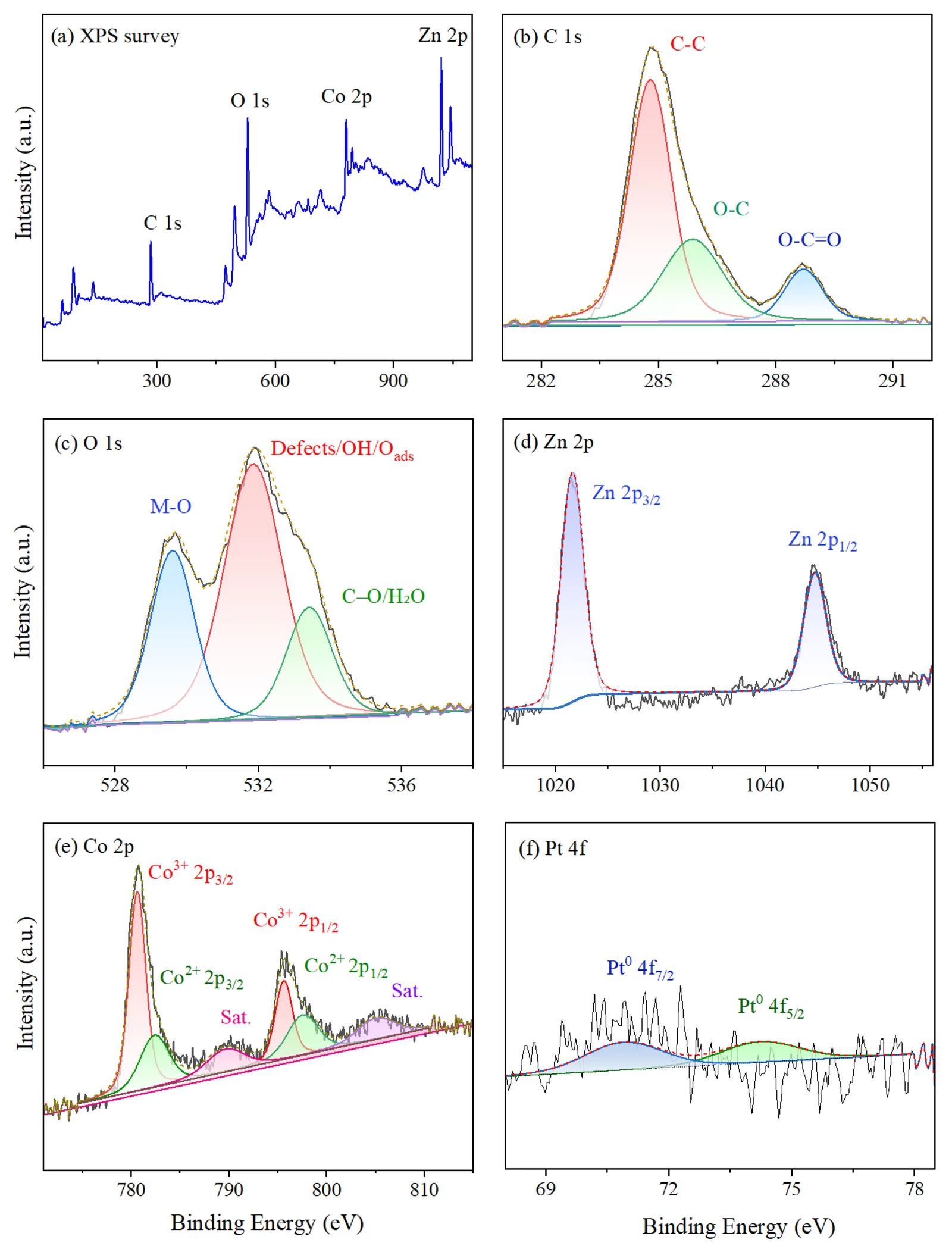
XPS analysis of the optimized ZC-0.5G composite: (**a**) survey spectrum and high-resolution spectra of (**b**) C 1s, (**c**) O 1s, (**d**) Zn 2p, (**e**) Co 2p, and (**f**) Pt 4f. The experimental spectra, fitted components, backgrounds, and cumulative fitted envelopes are displayed.

X-ray photoelectron spectroscopy was employed to examine the surface elemental composition and chemical states of the optimized ZC-0.5G composite. All binding energies were charge-corrected relative to the adventitious C 1s component at 284.8 eV [80]. The survey spectrum in Figure 7a shows signals corresponding to C, O, Zn, Co, and Pt, confirming the expected surface composition of the Pt-decorated ZC-G composite.

The high-resolution C 1s spectrum in Figure 7b was deconvoluted into components centered at 284.79, 285.87, and 288.71 eV. The dominant component at 284.79 eV is assigned to C–C/C=C bonds within the carbon framework, while the components at 285.87 and 288.71 eV are attributed to C–O and O–C=O species, respectively [41]. The oxygen-containing C 1s components are primarily associated with GO, whereas part of the graphitic carbon contribution may also originate from the carbon support of the Pt/C catalyst. These functional groups can serve as anchoring and interfacial-contact sites for oxide and Pt species; however, the XPS data alone do not establish a specific chemical bond between all components. Similar C 1s assignments have been reported for oxygen-functionalized graphene-based materials [81].

The O 1s spectrum in Figure 7c contains three components at 529.61, 531.87, and 533.43 eV. The low-binding-energy component at 529.61 eV is assigned to lattice metal-oxygen (M–O) bonds in the ZC spinel framework. The component at 531.87 eV is attributed to overlapping contributions from defect-associated oxygen, surface hydroxyl groups, and adsorbed oxygen species. The higher-binding-energy component at 533.43 eV is mainly associated with C–O-containing surface functionalities of GO and adsorbed H_2_O. Accordingly, the O 1s spectrum demonstrates the coexistence of lattice oxygen and chemically distinct surface oxygen species relevant to adsorption and surface redox processes during gas sensing [82,83].

The high-resolution Zn 2p spectrum in Figure 7d exhibits two characteristic peaks located at 1021.63 and 1044.73 eV, corresponding to the Zn 2p_3/2_ and Zn 2p_1/2_ states, respectively. The spin–orbit splitting of approximately 23.10 eV is characteristic of Zn^2+^ and agrees well with previously reported values for spinel ZC. These results confirm that zinc is present in the divalent oxidation state [84,85].

The Co 2p spectrum in Figure 7e shows the characteristic Co 2p_3/2_ and Co 2p_1/2_ envelopes, together with shake-up satellite features. The components at 780.64 and 795.63 eV are assigned to Co^3+^ 2p_3/2_ and Co^3+^ 2p_1/2_, respectively, whereas the components at 782.41 and 797.56 eV are attributed to Co^2+^ 2p_3/2_ and Co^2+^ 2p_1/2_, respectively. The satellite peaks centered at approximately 789.65 and 805.04 eV further support the multiplet character of the cobalt oxide surface. The coexistence of Co^2+^ and Co^3+^ confirms the mixed-valence surface chemistry of the cobalt-containing spinel. This redox-active surface may contribute to oxygen adsorption and gas-induced surface reactions; however, a direct quantitative relationship with the sensing response cannot be established from XPS alone. Mixed Co^2+^/Co^3+^ states and associated satellite features are widely observed in ZC spinel [84,86].

The Pt 4f spectrum in Figure 7f shows a weak but resolvable Pt 4f doublet, consistent with the low Pt loading in the composite. The Pt 4f_7/2_ and Pt 4f_5/2_ components are centered at 70.90 and 74.23 eV, respectively, giving a spin–orbit separation of 3.33 eV. Their fitted areas of 36.33 and 27.25, respectively, provide a Pt 4f_7/2_:Pt 4f_5/2_ area ratio of approximately 4:3, as expected for a physically consistent Pt 4f doublet. The binding energies are characteristic of predominantly metallic Pt^0^, rather than oxidized Pt species. Metallic Pt 4f_7/2_ and Pt 4f_5/2_ peaks near 70.9 and 74.2 eV have been independently reported for supported Pt catalysts [87]. The detection of metallic Pt supports its proposed catalytic role in H_2_ dissociation; nevertheless, the low signal-to-noise ratio indicates that the Pt oxidation-state interpretation should be restricted to the resolved fitted doublet and should not be extended to detailed quantitative surface-speciation analysis [88,89].

Overall, the XPS results confirm the presence of Zn^2+^, mixed Co^2+^/Co^3+^ states, lattice and surface-associated oxygen species, oxygen-functionalized carbon groups derived from GO, and predominantly metallic Pt^0^ on the optimized composite surface. These surface components provide the chemical basis for oxygen adsorption, oxide–GO interfacial interactions, and Pt-assisted H_2_ activation proposed in the sensing mechanism.

## 4. H_2_ Gas Sensing Performance

### 4.1. Influence of GO Loading on H_2_ Sensing Characteristics

The H_2_ sensing performance of the fabricated ZC-G sensors was evaluated against H_2_ concentrations of 500–10,000 ppm. Three sensor layers with different GO contents, denoted as ZC-0.1G, ZC-0.5G, and ZC-1G, were investigated to identify the optimal composition. The base resistance was strongly affected by GO loading and increased from 94.1 kΩ for ZC-0.1G to 12.37 and 12.26 MΩ for ZC-0.5G and ZC-1G, respectively. The increase in resistance can be attributed to the formation of additional ZC-G heterogeneous junctions and surface depletion regions and the lower conductivity of GO compared to metal oxide. These effects alter the charge transport pathways and increase the barrier for carrier migration across the sensor network [90,91].

**Figure 8:**
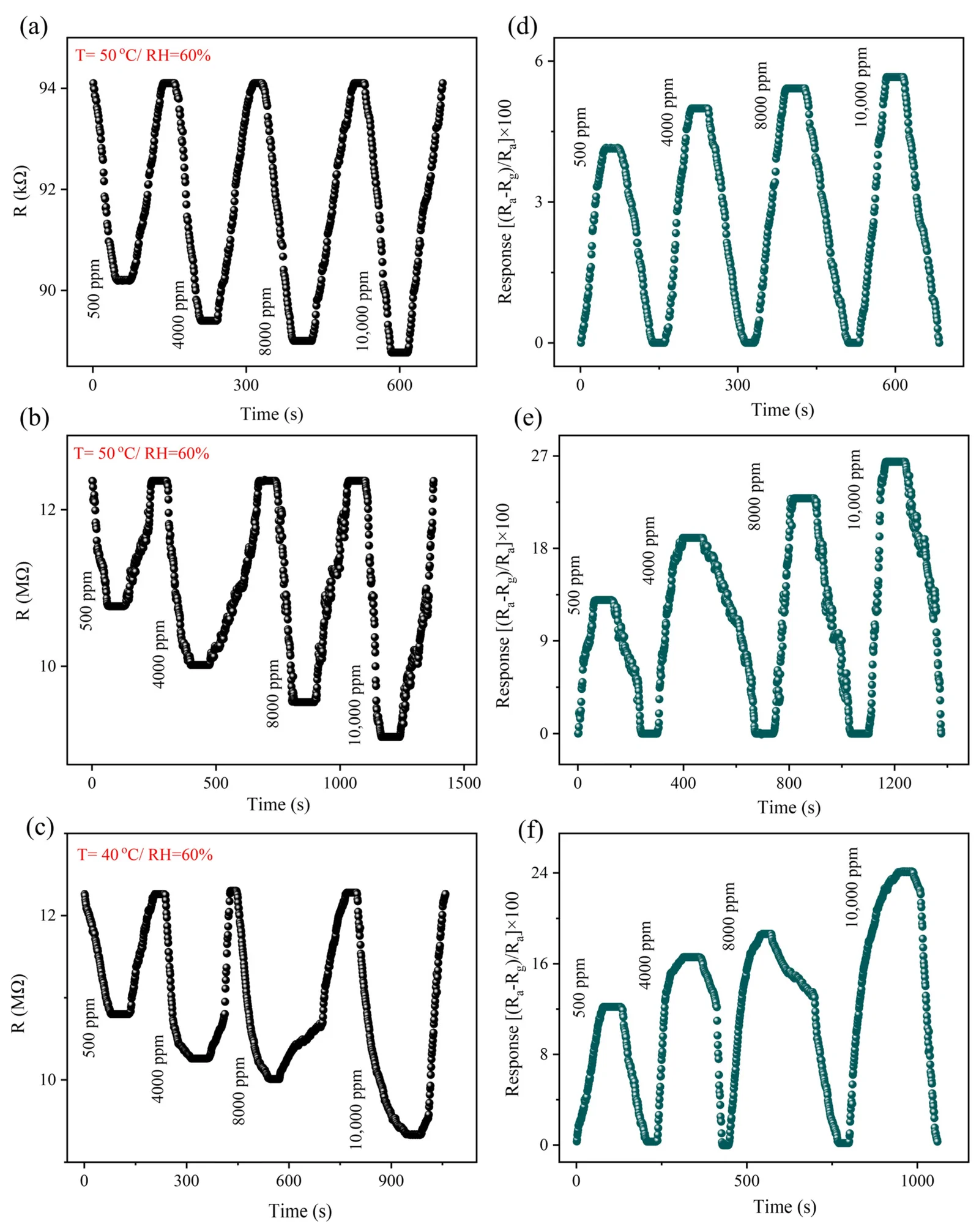
Dynamic resistance transients and corresponding sensing responses of (**a**,**d**) ZC-0.1G, (**b**,**e**) ZC-0.5G, and (**c**,**f**) ZC-1G toward 500–10,000 ppm H_2_. The operating temperature and RH for each measurement are indicated in the corresponding panels.

Figure 8a–c shows the dynamic resistance-time curves of the three sensors under successive cycles of exposure to H_2_ and N_2_. All sensors showed reversible resistance modulation upon H_2_ introduction and recovery upon gas removal, confirming the reproducible interaction between H_2_ molecules and the active sensor surface. The response of the layers increased with increasing H_2_ concentration, indicating concentration-dependent surface reaction kinetics. The corresponding response-time curves are presented in Figure 8d–f. The ZC-0.1G sensor showed limited responses of 4.14%, 4.99%, 5.42% and 5.66% for concentrations of 500, 4000, 8000 and 10,000 ppm H_2_, respectively. Increasing the GO content to 1 wt% significantly improved the response to 12.19, 16.58, 18.61 and 24.14% at the same concentrations. However, the highest response was obtained for the ZC-0.5G sensor with response values of 12.96%, 19.20%, 22.87%, and 26.43% from 500 to 10,000 ppm H_2_ concentrations, confirming that 0.5 wt% GO provides the most effective balance between surface accessibility, interfacial charge transfer, and gas diffusion pathways. The detailed sensing parameters, including response, t_res_, and t_rec_, for all sensing layers are summarized in Table 2. In addition to the improved response, the ZC-G composites generally showed shorter response and t_rec_ than the pristine ZC, indicating faster surface reaction kinetics and more efficient charge transfer. Among the sensing samples, ZC-0.5G provided a favorable balance between sensing response and dynamic performance.

The superior sensing performance of ZC-0.5G is closely correlated with its structural and textural characteristics. FESEM and TEM analyses revealed that the optimized composite preserves the hierarchical star–sphere architecture of ZC while establishing a homogeneous distribution of GO sheets and intimate oxide–carbon interfacial contact. Furthermore, BET measurements demonstrated that ZC-0.5G possesses the highest specific surface area (53.6 $m^{2}/g$) and pore volume (0.09 $cm^{3}/g$) among the investigated composites. The combination of a hierarchical dual-morphology structure, enlarged accessible surface area, and improved interfacial connectivity facilitates H_2_ diffusion, increases the density of active adsorption sites, and promotes more efficient charge transfer during gas exposure [10,92]. These structural advantages are reflected in the enhanced sensing response of ZC-0.5G compared with both pristine ZC and the other ZC-G composites.

**Figure 9:**
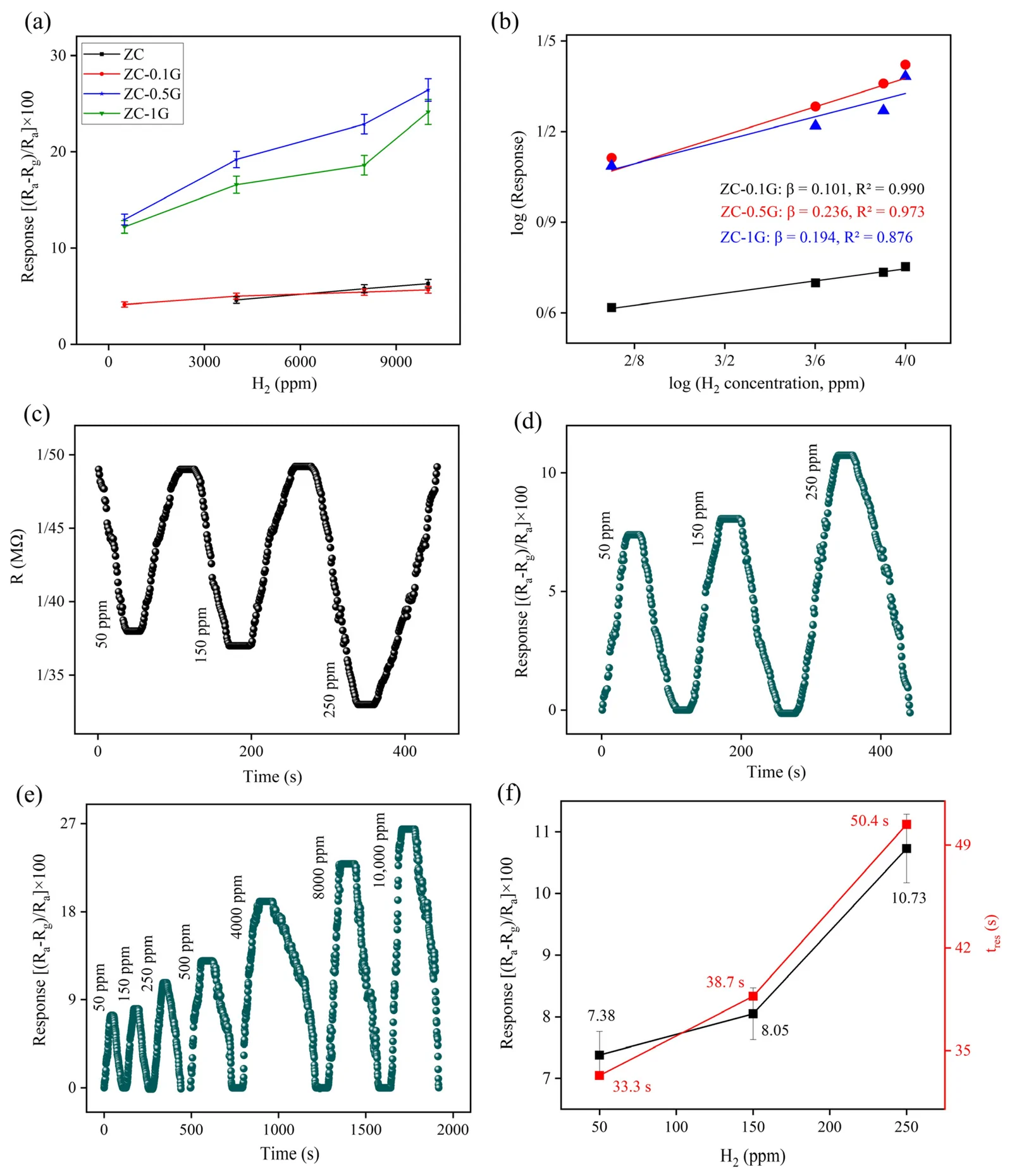
Concentration-dependent H_2_ sensing performance of ZC-G sensors: (**a**) response comparison of different GO loadings; (**b**) Logarithmic fitting of the sensor response as a function of H_2_ concentration for ZC-0.1G, ZC-0.5G, and ZC-1G sensing layers. (**c**,**d**) resistance and response curves of ZC-0.5G at 50–250 ppm H_2_; (**e**) response over 50–10,000 ppm H_2_; and (**f**) response and t_res_ versus H_2_ concentration. Error bars in panels (**a**,**f**) represent the standard deviation of three repeated measurements performed using the same sensing layer (n=3).

For comparison purposes, pristine ZC was also evaluated as a sensing material and its sensing performance is compared with the ZC-G layers in Figure 9a, while the corresponding sensing parameters are included in Table 2. The pristine ZC showed no detectable response at 500 ppm H_2_ and only limited responses of 4.60%, 5.78% and 6.30% toward 4000, 8000 and 10,000 ppm H_2_, respectively. Furthermore, significantly longer t_res_/t_rec_ were observed compared to composite sensors. Such behavior is usually associated with the limited density of active adsorption sites, slower surface reaction kinetics and the absence of conductive interfacial pathways and catalytic activation centers, which limit efficient charge transfer and hydrogen dissociation at relatively low operating temperatures [10,93].

Figure 9b shows the logarithmic fit of the response of the sensing layers as a function of H_2_ concentration. An almost linear relationship between log (Response) and log (H_2_ concentration, ppm) for all the sensing layers indicates that the sensing behavior follows a power-law relationship depending on the concentration. The slope (β) values obtained for ZC-0.1G, ZC-0.5G, and ZC-1G are 0.101, 0.236, and 0.194, respectively. Among the investigated samples, ZC-0.5G with the highest β value along with a high fitting coefficient (R^2^ = 0.973) shows the strongest dependence of the response on H_2_ concentration. This behavior indicates a more effective balance between active adsorption sites, interfacial charge transfer and gas diffusion pathways and is the result of the concentration sensitivity to H_2_.

To evaluate the low-concentration sensing capability of the optimized sensor, ZC-0.5G was further tested toward 50, 150, and 250 ppm H_2_, as shown in Figure 9c–f. The sensor produced clear and detectable responses of 7.38%, 8.05%, and 10.73% with t_res_ of 33.3, 38.7, and 50.4 s, respectively. A gradual increase in t_res_ with increasing H_2_ concentration was also observed, which can be attributed to the larger number of H_2_ molecules participating in the surface reaction. These results demonstrate the ability of the optimized ZC-0.5G layer to detect low H_2_ concentrations with stable and concentration-dependent behavior. Observation of the response at concentrations as low as 50 ppm shows that the sensor signal is significantly above the baseline oscillation level, demonstrating the sensor’s ability to detect H_2_ at low concentrations. The improved sensing performance of ZC-0.5G can be attributed to the synergistic contribution of hierarchical ZC, GO, and Pt nanoparticles. The hierarchical star–sphere architecture enhances surface accessibility and gas diffusion, while GO improves interfacial charge transfer and prevents severe particle aggregation at optimal loading. Pt nanoparticles act as catalytic sensitizers, enhancing H_2_ dissociation and accelerating surface reactions through chemical sensitization. The superior sensing performance of ZC-0.5G suggests that 0.5 wt% GO provides the best balance between surface accessibility, gas diffusion pathways, oxide–GO interfacial contact, Pt-assisted H_2_ activation, and charge transport. Low GO loading provides insufficient interfacial modulation, while excessive GO coverage can partially shield the active oxide sites and hinder gas access. As a result, the optimal composition of ZC-0.5G provides an efficient sensing interface in which gas diffusion, charge transfer and hydrogen activation are simultaneously enhanced, leading to the highest H_2_ response among the sensors investigated.

### 4.2. Effect of Operating Temperature and RH on H_2_ Sensing Performance

**Figure 10:**
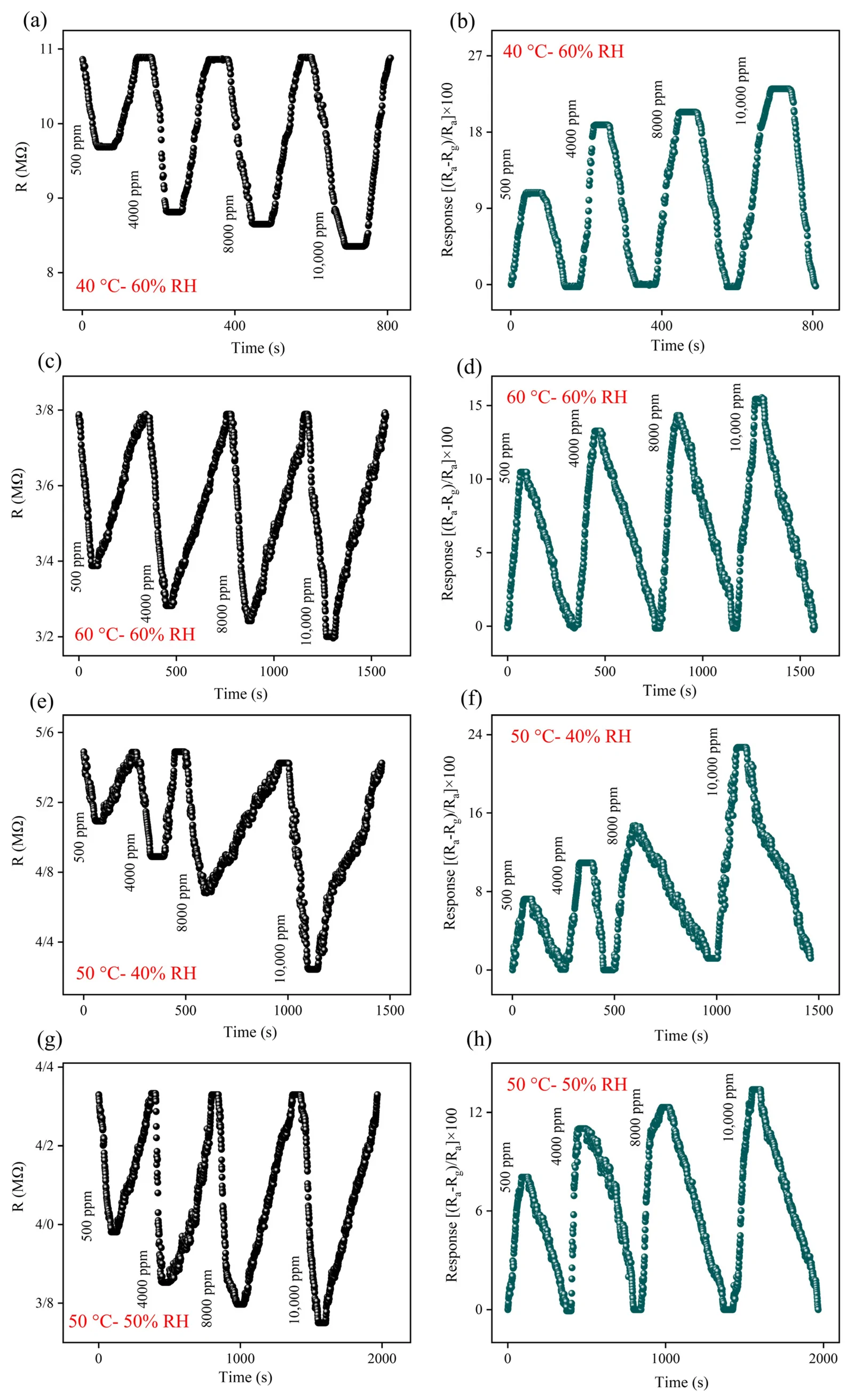
Dynamic resistance transients and corresponding responses of ZC-0.5G toward 500–10,000 ppm H_2_: measurements at different temperatures and RH levels: (**a**,**b**) 40 °C, 60% RH; (**c**,**d**) 60 °C, 60% RH; (**e**,**f**) 50 °C, 40% RH; (**g**,**h**) 50 °C, 50% RH. Panels (**a**,**c**,**e**,**g**) show resistance transients, and panels (**b**,**d**,**f**,**h**) show the corresponding responses.

**Figure 11:**
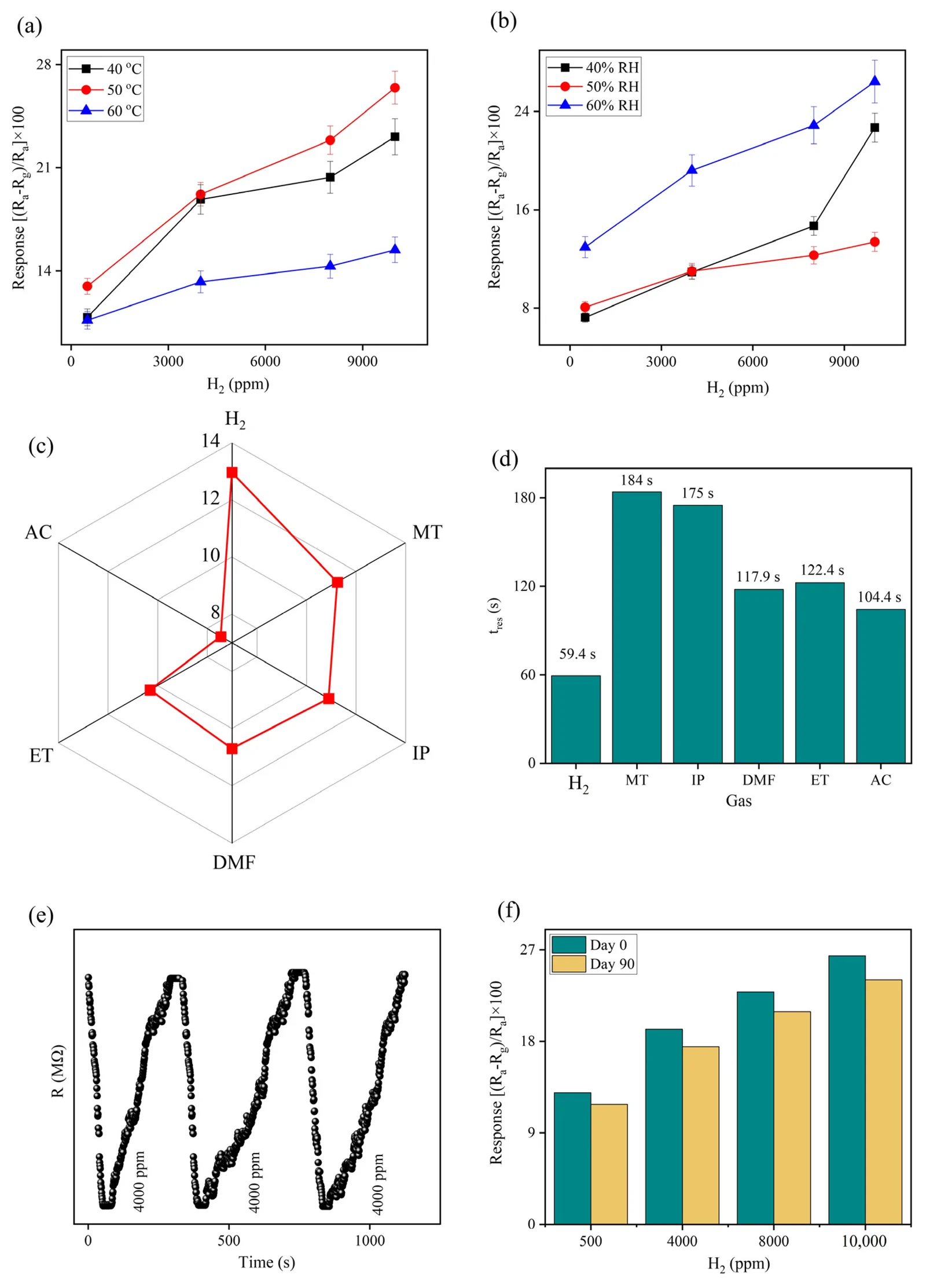
(**a**) Effect of operating temperature and (**b**) effect of RH on the H_2_ sensing response. (**c**) H_2_ sensing selectivity and (**d**) comparison of t_res_ toward different gases. (**e**) repeatability of the optimized ZC-0.5G sensor toward 4000 ppm H_2_. (**f**) Comparison of H_2_ sensing responses of the ZC-0.5G before and after 90 days of storage. Error bars in panels (**a**,**b**) represent the standard deviation of three repeated measurements performed using the same sensing layer (n=3).

To evaluate the environmental robustness of the optimized ZC-0.5G sensor, the H_2_ sensing behavior was investigated under different operating temperatures and RH. The dynamic resistance and response transients obtained under these conditions are presented in Figure 10, while the corresponding sensing parameters are summarized in Table 3 and presented in Figure 11 for comparison.

The effect of operating temperature was first investigated at a constant of 60% RH. As shown in Figure 10a–d and Figure 11a, the sensor exhibited reversible and concentration-dependent responses to H_2_ concentrations from 500 to 10,000 ppm at all temperatures investigated. At 40 °C, the response increased from 10.86% to 23.10% as the H_2_ concentration increased from 500 to 10,000 ppm. Increasing the operating temperature to 50 °C further enhanced the sensing performance, resulting in the highest response values over the entire concentration range, reaching 26.43% at 10,000 ppm H_2_. However, further increasing the temperature to 60 °C resulted in a significant decrease in response, with the maximum value at the same gas concentration decreasing to 15.43%.

The observed temperature dependence reflects the balance between H_2_ adsorption and desorption, the coverage and reactivity of oxygen-containing surface species, the dissociation of H_2_ by Pt, and the kinetics of subsequent surface redox reactions. At 40 °C, the lower thermal energy limits the rate of H_2_ activation and surface reaction, resulting in lower resistance modulation. Increasing the temperature to 50 °C accelerates these processes while maintaining sufficient surface residence time and coverage of the adsorbed species, resulting in the highest response in the temperature range studied. At 60 °C, the accelerated desorption and shorter residence time of the reactive surface species reduce the effective surface coverage and thus the response. Therefore, 50 °C was considered as the optimum temperature within the experimentally investigated temperature range of 40–60 °C, rather than as an absolute operating temperature limit for the material [45]. Room-temperature operation of the optimized ternary ZC-0.5G sensor was beyond the scope of the present study. Therefore, although it remains an important objective for practical H_2_ sensors, no conclusion regarding room-temperature sensing performance can be drawn from the present results [12,94].

Subsequently, the effect of RH was evaluated at a constant operating temperature of 50 °C for the ZC-0.5G sensing layer. The 60% RH condition corresponded to the typical laboratory ambient humidity during the measurements and was used as the reference condition, while additional experiments were performed at 40% and 50% RH to quantify the humidity-dependent changes. As shown in Figure 10e–h and Figure 11b, the sensor maintained reversible and H_2_ concentration-dependent responses at all investigated RH levels. However, the response amplitude and recovery behavior varied with humidity. At a concentration of 10,000 ppm H_2_, the responses were 22.69%, 13.39% and 26.43% at 40%, 50% and 60% RH, respectively. This non-uniform trend suggests that humidity does not have a purely inhibitory or enhancing effect. Adsorbed water can change the surface hydroxylation and surface charge state, while the oxygen-containing functional groups of GO provide additional sites for water interaction. At the same time, water molecules may compete with H_2_ and oxygen-containing species for the available adsorption sites. Therefore, the measured response results from a balance between surface hydroxylation, competitive adsorption, and humidity-dependent modulation of the surface resistance. Accordingly, 60% RH represents the condition that produced the highest response among the RH levels investigated. Humidity interference in metal oxide H_2_ sensors is strongly dependent on the surface chemistry and morphology and therefore needs to be evaluated and calibrated for the intended operating environment [95,96,97].

Overall, the superior sensing performance observed at 50 °C and 60% RH can be directly attributed to the structural features of the optimized ZC-0.5G composite. BET analysis revealed that this sample has the highest specific surface area and pore volume among the investigated compounds, which provides abundant active adsorption sites and efficient diffusion pathways for H_2_ molecules. Morphological observations also revealed a well-preserved hierarchical star–sphere architecture that is uniformly integrated with GO sheets and forms numerous interfacial connections that facilitate charge transfer. In addition, the Pt nanoparticles act as catalytic enhancers by enhancing H_2_ dissociation and accelerating surface redox reactions. Therefore, the combined contribution of hierarchical porosity, efficient interfacial charge transfer, and catalytic surface activation enables efficient resistance modulation and improved H_2_ sensing under optimal environmental conditions.

### 4.3. Selectivity and Cycling Stability of the Optimized ZC-0.5G Sensor

The selectivity of the optimized ZC-0.5G sensor towards 500 ppm of several interfering volatile compounds, including methanol (MT), isopropanol (IPA), dimethylformamide (DMF), EtOH and acetone (AC), was evaluated under the same operating conditions (50 °C and 60% RH). The sensor responses and corresponding t_res_ are presented in Figure 11c,d. As shown in Figure 11c, the sensor showed the highest response towards H_2_, reaching a value of 12.96%. Lower responses were obtained for MT (11.25%), IPA (10.90%), DMF (10.70%), EtOH (10.30%) and AC (7.45%). Although several VOCs produced measurable signals, H_2_ showed the highest response among all gases tested, confirming the preferential interaction of this sensing layer with H_2_ molecules. More importantly, a significant difference in the response kinetics was observed (Figure 11d). The t_res_ for H_2_ was only 59.4 s, while significantly longer t_res_ were measured for MT (184 s), IPA (175 s), DMF (117.9 s), and EtOH (122.4 s). Thus, H_2_, in addition to showing the highest response, also produced the fastest sensing dynamics. This combination of improved response and accelerated kinetics provides another criterion for reliably distinguishing H_2_ from other interfering gases. The preferential sensing behavior toward H_2_ can be attributed to several factors. First, H_2_ has the smallest molecular diameter (≈0.289 nm) among the gases studied, which allows for rapid diffusion through the porous architecture and efficient access to active adsorption sites. Second, Pt nanoparticles dispersed on the composite surface enhance the catalytic dissociation of H_2_ into highly reactive atomic hydrogen species via a spillover mechanism. These dissociated species readily react with oxygen ions chemisorbed on the ZC surface, causing significant charge transfer and resistance modulation. In contrast, larger VOC molecules require more complex surface oxidation pathways and exhibit slower diffusion and reaction kinetics, leading to longer t_res_ and weaker effective resistance changes [12].

To further investigate, the repeatability of the optimized sensor was tested through repeated sensing cycles at a concentration of 4000 ppm H_2_, as shown in Figure 11e. Almost equal response values were obtained in successive cycles, without significant changes in the base resistance or response amplitude. The excellent repeatability confirms the stability of the adsorption–desorption processes and demonstrates the robustness of the ZC-0.5G sensing layer under repeated H_2_ exposure. Such stable cyclic behavior demonstrates the efficient recovery of active surface sites and highlights the suitability of the sensor for practical applications.

### 4.4. Long-Term Stability of the Optimized ZC-0.5G Sensor

To evaluate the long-term stability of the optimized sensing layer, the ZC-0.5G sensor was re-evaluated after 90 days of storage in laboratory ambient conditions toward different concentrations of H_2_ under the same operating conditions. As summarized in Table 4 and Figure 11f, the sensor retained approximately 91% of its initial response after the storage period, demonstrating the good long-term functional stability of the optimized sensing layer.

### 4.5. Morphological and Sensing Evaluation of the Control Composites

To distinguish the individual contributions of GO and Pt to the H_2_ sensing behavior, control composites ZC-G and ZC-Pt were prepared using the same synthesis and sensor layer fabrication methods as applied to the corresponding components in the ternary composites. FESEM analysis was first performed to verify whether the characteristic hierarchical morphology of ZC was preserved after the separate incorporation of GO and Pt.

**Figure 12:**
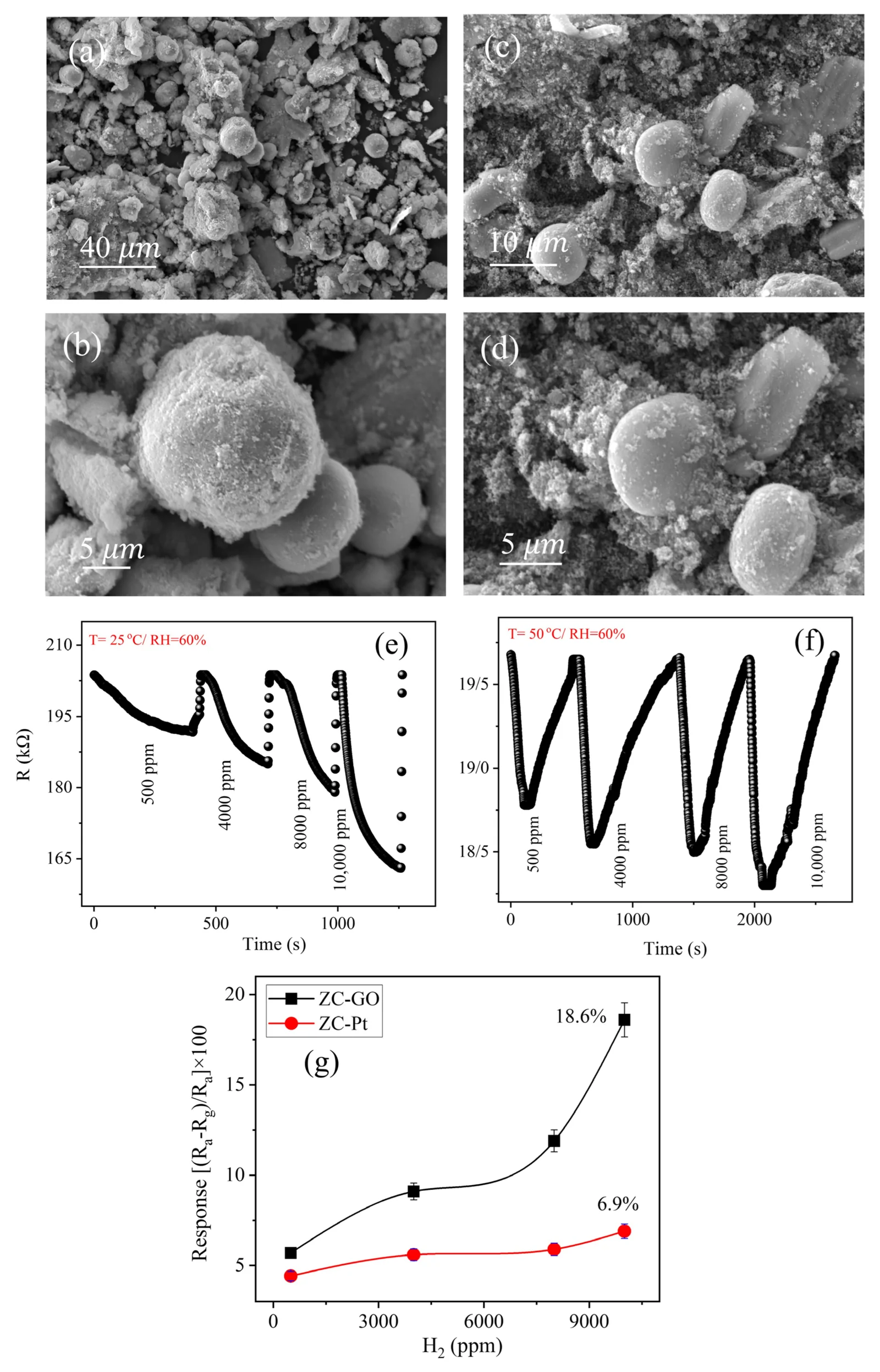
FESEM images of the control composites: (**a**,**b**) ZC–GO and (**c**,**d**) ZC–Pt; dynamic H_2_ sensing transients (**e**) ZC–GO and (**f**) ZC–Pt; and (**g**) comparison of the sensing responses of the two control composites toward different H_2_ concentrations. Error bars in panels (**g**) represent the standard deviation of three repeated measurements performed using the same sensing layer (n=3).

The FESEM images of ZC–G at different magnifications (Figure 12a,b) reveal the coexistence of microspherical particles and irregular star-like hierarchical aggregates. The microspheres exhibit comparatively rough surfaces composed of closely assembled fine particles, while the star-like domains remain distinguishable within the surrounding agglomerated oxide matrix. Although the individual GO sheets cannot be unambiguously distinguished by FESEM, the preservation of both morphological components indicates that GO incorporation preserves the characteristic hierarchical morphology of ZC. The heterogeneous particle packing and interparticle voids also produce an open, texturally rough structure that may facilitate gas diffusion within the sensing layer.

The ZC–Pt control composite (Figure 12c,d) similarly retains the dual morphology, comprising rounded microspherical particles together with preserved star-like hierarchical structures. Compared with ZC–GO, several microspheres exhibit relatively smoother and more compact outer surfaces, whereas fine particulate aggregates remain distributed around and between the larger structures. Individual Pt nanoparticles cannot be resolved at the available FESEM magnification because of the low Pt loading; therefore, the images primarily confirm that the Pt incorporation process preserves the overall star–sphere framework without altering the characteristic hierarchical architecture of ZC.

The H_2_-sensing performances of the ZC–GO and ZC–Pt control composites were subsequently evaluated to distinguish the individual contributions of GO and Pt to the sensing behavior (Figure 12e–g). The ZC–GO and ZC–Pt control sensors were evaluated at room temperature and 50 °C, respectively, under the same RH of 60%, corresponding to the conditions at which stable and measurable sensing responses were obtained. Both control samples exhibited stable and reversible resistance changes upon repeated H_2_ exposure, demonstrating that each component individually contributes to the sensing process. The ZC–GO sample showed a more pronounced concentration-dependent increase in response, particularly at higher H_2_ concentrations, indicating that GO effectively enhances the concentration-dependent sensing response, likely through improved interfacial charge transport and enhanced surface accessibility. In contrast, the ZC–Pt sample exhibited comparatively lower response values but substantially faster response kinetics, reflecting the catalytic role of Pt in promoting H_2_ dissociation and accelerating the surface reaction. Conversely, although the ZC–GO control exhibited a considerably higher sensing response, its response kinetics were markedly slower than those of ZC–Pt, further demonstrating the distinct functional roles of GO and Pt during H_2_ sensing. Under the tested control conditions, neither control sample reproduced the combined high response and favorable kinetics observed for the ternary ZC–GO–Pt composite. These results support the superior H_2_-sensing characteristics originate from the synergistic combination of GO-assisted charge transport, Pt-catalyzed surface activation, and the hierarchical ZC architecture rather than from the individual contribution of either GO or Pt alone.

These control experiments provide direct experimental support for the complementary functions proposed for GO and Pt. While GO primarily enhances the sensing response through improved interfacial charge transport and gas accessibility, Pt predominantly accelerates the surface reaction kinetics via catalytic H_2_ dissociation. The combination of these complementary effects in the ternary composite therefore results in a synergistic enhancement that cannot be achieved by either component individually.

Table 5 compares the H_2_ sensing performance of the current sensor with previously reported graphene-based and spinel-based sensing materials. Although the reported studies were conducted under different operating temperatures, hydrogen concentrations, and response definitions, the ZC-0.5G sensor exhibits competitive sensing characteristics. In particular, the sensor achieves a relatively high response at a relatively low operating temperature of 50 °C while maintaining a t_res_ comparable to many previously reported spinel-based systems. Overall, the comparison demonstrates a competitive balance between sensing response, response kinetics, and operating temperature. More importantly, the significance of the present platform lies in the integrated structural, interfacial, and catalytic design of the sensing layer, supported by systematic evaluation under different H_2_ concentrations and environmental conditions.

## 5. Schematic Illustration and Discussion of the H_2_ Sensing Mechanism

The H_2_ sensing mechanism in the ZC-G sensor is controlled by the synergistic contributions of surface oxygen chemisorption, catalytic H_2_ dissociation on Pt nanoparticles, interfacial charge transfer, and heterojunction-induced resistance modulation within the oxide–graphene hybrid.

**Figure 13:**
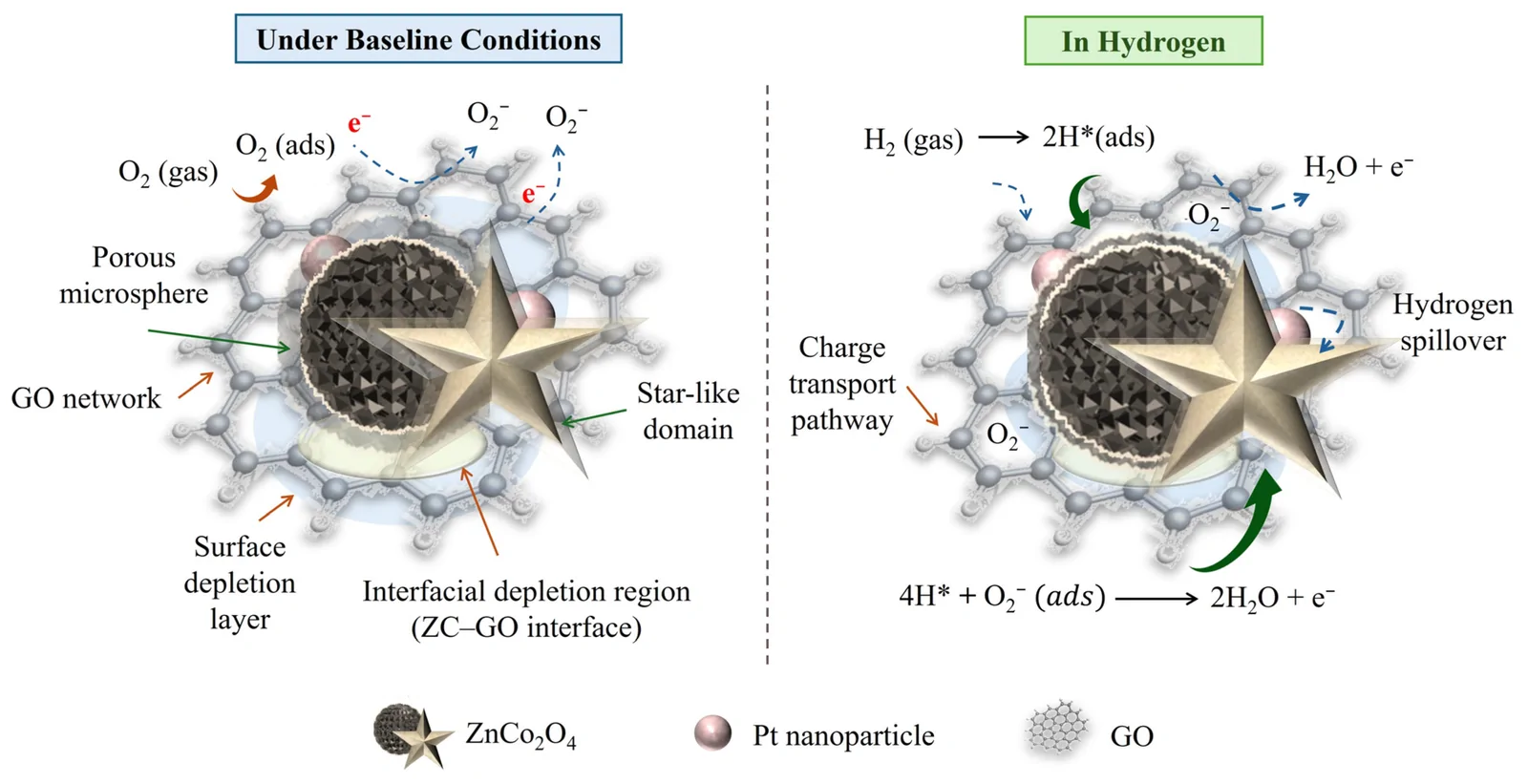
Schematic illustration of the H_2_ sensing mechanism in the Pt-decorated ZC−G composite toward H_2_.

As schematically illustrated in Figure 13, the sensing response originates from charge carrier transport processes occurring at the surface and interfaces of the sensing layer, which dynamically regulate the depletion region thickness and consequently modulate the overall electrical conductivity of the composite [104]. Under the baseline atmosphere, oxygen species pre-adsorbed on the ZC/Pt surface participate in the sensing reaction, while N_2_ was used as the carrier/purge gas during recovery [97]. At the operating temperatures investigated (40–60 °C), $O_{2}^{-}$ is expected to be the dominant chemisorbed oxygen species, while a small amount of more reactive oxygen species may be present at the Pt-assisted active sites [96].

The electron withdrawal from the near-surface region leads to the formation of a surface electron depletion layer in the ZC. Simultaneously, electronic equilibration at the ZC–G interface forms an interfacial depletion region, which provides an additional resistance modulation pathway during H_2_ exposure.

O_2_ (gas) → O_2_ (ads) (2)

(3) $$O_{2}\;(\mathrm{ads})+e^{-}\;\to \;O_{2}^{-}(\mathrm{ads})$$

Pt contributes through both chemical and electronic sensitization mechanisms, accelerating hydrogen oxidation kinetics and enhancing the resistance modulation at the metal-semiconductor interface. After the introduction of H_2_ gas, hydrogen molecules are efficiently dissociated into highly reactive atomic hydrogen species (H*) on Pt nanoparticles owing to their strong catalytic activity and low activation energy barrier [88]. The generated H* species rapidly migrate toward the oxide surface through a spillover-assisted pathway and react with the adsorbed oxygen ions on both Pt and ZC surfaces, forming H_2_O molecules and releasing trapped electrons back into the conduction channel [105,106]:

H_2_ (gas) → 2H* (ads) (4)

(5) $$4H*\;(\mathrm{ads})+O_{2}^{-}(\mathrm{ads})\;\to \;2H_{2}O+e^{-}$$

Consequently, the reaction between hydrogen and the chemically adsorbed oxygen species returns electrons to the sensing layer, leading to a reduction in the thickness of the depletion layer and a corresponding decrease in the sensor resistance. The catalytic spillover of hydrogen from the Pt to the oxide surface significantly accelerates these reactions, explaining the increased sensitivity and faster response dynamics observed in the ZC-G sensor [107]. In addition to chemical sensitization, electronic sensitization plays an important role at the Pt/ZC interface. The adsorbed hydrogen species can modify the local electronic state of the supported Pt nanoparticles and the surrounding Pt/C–oxide contact regions, thereby contributing to the observed resistance modulation [108].

Furthermore, the hierarchical star–sphere dual architecture offers an additional structural advantage. The microsphere domains act as porous reservoirs for gas adsorption and diffusion, while the star-like protrusions expose abundant edge sites, corners, and high-energy facets that act as preferential reaction centers. The coexistence of these complementary morphological domains facilitates rapid gas transport, increases the density of accessible active sites, and promotes efficient charge exchange across the sensing layer [109,110]. In addition, the ZC–G heterojunction provides an additional resistance modulation pathway through dynamic variation in the interfacial depletion region during H_2_ exposure [111]. GO sheets create interfacial charge transfer pathways, suppress particle aggregation, and create extended oxide–carbon contact areas with ZC, thereby increasing the density of interfacial sites involved in resistance modulation.

In conclusion, the outstanding sensing behavior of the optimized ZC-0.5G composite arises from the synergistic integration of the hierarchical dual star–sphere architecture of ZC that provides abundant adsorption and reaction sites, GO-mediated interfacial coupling and efficient charge transfer pathways, and Pt-induced chemical and electronic sensitization. The simultaneous coupling of morphology engineering, GO-mediated interface modulation, and Pt-assisted catalytic sensitization creates an efficient H_2_ sensing platform with improved response under the investigated temperature and humidity conditions [112,113].

## 6. Conclusions

In this work, hierarchical ZC-G nanocomposites with different GO contents were successfully synthesized and investigated as H_2_ sensing materials. This study emphasizes the importance of integrating dual hierarchical morphology, optimized carbon coupling, and catalytic sensitization into a single metal oxide/carbon hybrid platform. The main findings are summarized as follows:

- XRD analysis confirmed the formation of cubic spinel phase of ZC without any detectable secondary oxide phase after addition of GO and decoration with Pt.
- FESEM and TEM images revealed a dual hierarchical architecture of ZC consisting of porous microspheres and star-shaped assemblies. This star–sphere structure provides a multi-scale morphology that can simultaneously support gas diffusion, active site exposure, and surface contact with GO sheets.
- BET analysis showed that ZC-0.5G had the highest specific surface area (53.6 m^2^/g) and pore volume (0.09 cm^3^/g), supporting the role of the optimized architecture in increasing accessible surface area and improving pore accessibility/gas diffusion pathways.
- Among the investigated sensing layers, ZC-0.5G showed the best H_2_ sensing performance with responses of 12.96%, 19.20%, 22.87%, and 26.43% for concentrations of 500, 4000, 8000, and 10,000 ppm H_2_, respectively.
- The ZC-0.5G sensor was able to detect low H_2_ concentrations down to 50 ppm and produced a clear response of 7.38%, demonstrating its potential for low H_2_ levels.
- Sensing measurements performed at temperatures of 40–60 °C and 40–60% RH showed that the highest response among the conditions investigated was obtained at 50 °C and 60% RH. The sensor maintained reversible sensing behavior throughout the investigated range, although the magnitude of the response and recovery characteristics were dependent on RH.
- Selectivity testing showed a preferential response to H_2_ compared to MT, IPA, EtOH, AC, and DMF. Although the interfering gases produced measurable responses, H_2_ showed the highest response and faster response kinetics, supporting its preferential detection.
- Repeatability tests confirmed repeatable sensing behavior over successive cycles, indicating reversible gas–surface interactions and operational stability of the optimized sensing layer.
- Long-term stability evaluation showed that the optimized ZC-0.5G sensor retained approximately 91% of its initial sensing response after 90 days of storage, indicating good durability and stability of the sensor architecture.

This study provides a systematic investigation of the H_2_ sensing behavior of ZC-G-based composites, including the effects of GO loading, H_2_ concentration, operating temperature, humidity, selectivity, reproducibility, and storage stability. The results establish a clear correlation between composite architecture, surface properties, and sensing performance, highlighting the critical role of optimal metal oxide–GO integration in achieving efficient H_2_ detection.

The superior sensing performance of the optimized ZC-0.5G sensor is attributed to the synergistic interaction between the hierarchical ZC architecture, GO-mediated interfacial charge transfer, and Pt-assisted catalytic activation. The ZC–GO and ZC–Pt control experiments further support the complementary contributions of GO and Pt, with GO predominantly enhancing the sensing response and Pt promoting faster H_2_ response kinetics. This combination enables improved response, reliable detection at low concentrations, and stable sensing behavior under operational conditions. Overall, this work demonstrates that the integration of hierarchical ZC structures with GO and Pt nanoparticles provides an effective strategy for the development of high-performance H_2_ sensors. These findings provide valuable guidance for the design of next-generation metal oxide/carbon hybrid sensors in which morphology engineering, interface modulation, and catalytic sensitization are simultaneously optimized.

## 7. Limitations and Future Perspectives

Although the proposed sensor showed promising H_2_ sensing performance, several aspects require further investigation. First, the sensing behavior at sub-ppm H_2_ concentrations has not been investigated and needs to be evaluated for early leak detection applications. Second, long-term stability beyond the current test period and performance under continuously varying humidity conditions need to be investigated. Room-temperature sensing performance also remains to be experimentally evaluated, together with RH-dependent calibration and real-time humidity compensation under dynamically changing environmental conditions. Third, in situ spectroscopic investigations and theoretical calculations may provide deeper insights into the interfacial charge transfer mechanism and catalytic role of Pt nanoparticles. Future studies may also focus on flexible sensor platforms, noble metal optimization, and advanced oxide/graphene heterostructures to further improve the sensitivity, selectivity, and response kinetics under real-world operating conditions.

## Figures and Tables

**Table 1:** BET surface area, total pore volume and average pore size of the ZC-G composites.

Samples	$S_{BET}(m^{2}/g)$	Pore Volume $\left(cm^{3}/g\right)$	Average Pore Size (nm)
ZC-0.1G	26	0.01	12.22
ZC-0.5G	53.6	0.09	14.19
ZC-1G	38	0.01	14.5

**Table 2:** Response, t_res_, and t_rec_ of ZC-G sensors with different GO loadings toward various H_2_ concentrations.

H_2_ (ppm)	Sample	Response (%)	t_res_ (s)	t_rec_ (s)
500	ZC	-	-	-
	ZC-0.1G	4.14	42.3	57.6
	ZC-0.5G	12.96	59.4	99.9
	ZC-1G	12.19	72	81
4000	ZC	4.6	98	174.6
	ZC-0.1G	4.99	44.1	63.9
	ZC-0.5G	19.2	84.6	113.7
	ZC-1G	16.58	70.2	60.3
8000	ZC	5.78	135.1	285
	ZC-0.1G	5.42	54	72.9
	ZC-0.5G	22.87	63.9	123.3
	ZC-1G	18.61	81	176.4
10,000	ZC	6.3	170.7	312
	ZC-0.1G	5.66	45.9	60.3
	ZC-0.5G	26.43	55.8	125.5
	ZC-1G	24.14	120.6	72.2

**Table 3:** H_2_ sensing parameters of the optimized ZC-0.5G sensor under different temperature and RH conditions.

Condition	H_2_ (ppm)	Response (%)	t_res_ (s)	t_rec_ (s)
40 °C, 60% RH	500	10.86	35.1	54.9
	4000	18.85	41.4	70.2
	8000	20.35	70.23	87.3
	10,000	23.1	96.3	79.2
60 °C, 60% RH	500	10.65	58.5	208
	4000	13.26	73.8	249.3
	8000	14.32	74.2	253.8
	10,000	15.43	83.7	228.6
50 °C, 40% RH	500	7.23	52.2	124.2
	4000	10.92	62.1	47.7
	8000	14.69	83.7	297.9
	10,000	22.69	86.4	282.6
50 °C, 50% RH	500	8.06	77.4	216
	4000	11.01	39.6	270.9
	8000	12.3	26.1	309.6
	10,000	13.39	108.9	327.6

**Table 4:** Long-term stability of the optimized ZC-0.5G sensor after 90 days of storage.

H_2_ (ppm)	Response Day 0	Response Day 90	t_res_ Day 0 (s)	t_res_ Day 90 (s)
500	12.96	11.80	59.4	64.0
4000	19.20	17.47	84.6	91.0
8000	22.87	20.81	63.9	69.0
10,000	26.43	24.05	55.8	60.0

**Table 5:** Comparison of the H_2_ sensing performance of the present ZC-G sensor with representative graphene-based and metal oxide/graphene hybrid H_2_ sensors reported in the literature.

Material	H_2_ (ppm)	Response	t_res_ (s)	Temp. (°C)	Ref.
CeO_2_-Fe_2_O_3_-GO	40	1	8	RT	[98]
rGO-MnCo_2_O_4_	250	12.77%	9	RT	[99]
Ppy-ZnMn_2_O_4_	15,000	1.34	102	RT	[100]
Ppy-ZnMn_2_O_4_	15,000	1.23	125.1	RT	[101]
Pd -Zn_2_SnO_4_-ZnO	1000	15.6	120–240	400	[102]
Pd-rGO–ZnFe_2_O_4_	200	11.43%	18	100	[103]
ZC-0.5G	500	12.96%	59.4	50	This work
ZC-0.5G	10,000	26.43%	55.8	50	This work

The response values reported in Table 5 were adopted from the original references and are based on the response definitions used in the respective studies, including S = (R_a_ − R_g_)/R_g_ × 100 or S = R_a_/R_g_.

## Data Availability

The data that support the findings of this study are available within the article. Raw data are available from the corresponding author upon reasonable request.
